# Metabolic engineering of *Corynebacterium glutamicum* for high-yield de novo biosynthesis of 5-aminovaleramide, a promising bio-based monomer

**DOI:** 10.1186/s12934-026-02922-1

**Published:** 2026-02-02

**Authors:** Annalena Sommer, Sarah Pauli, Michael Kohlstedt, Judith Becker, Christoph Wittmann

**Affiliations:** Institute for Systems Biotechnology, Saarland University, Saarbrücken, Germany

**Keywords:** 5-Aminovaleramide, *Corynebacterium glutamicum*, Redox engineering, GapN, Lysine 2-monooxygenase, Metabolic engineering, Bio-based monomers, Amino-acid-derived chemicals

## Abstract

**Background:**

Lysine-derived C5 compounds are important intermediates in cellular metabolism and promising building blocks for sustainable polymer chemistry. Among these, 5-aminovalerate (5-AVA) has been extensively studied as a platform chemical produced via a two-step microbial pathway. However, its direct precursor, 5-aminovaleramide (5-AVD), generated from lysine by lysine 2-monooxygenase, remains largely unexplored. Notably, 5-AVD is an attractive product in its own right, as it provides a versatile intermediate for the synthesis of polyamides and other nitrogen-containing chemicals. Here, we establish the first de novo microbial production of 5-AVD by systematically engineering *Corynebacterium glutamicum* for optimized precursor flux, product export, and redox balance.

**Results:**

Trace secretion of 5-AVD was discovered in 5-AVA-producing strains, and tolerance studies showed that *C. glutamicum* can withstand high 5-AVD concentrations. To exploit this trait, the lysine-producing strain LYS-12 was engineered to express the *davB* gene from *Pseudomonas putida* under the constitutive *tuf* promoter, resulting in increased 5-AVD secretion. Pathway analysis revealed that the native exporter LysE is essential for efficient 5-AVD export, while heterologous GABA permeases provided no benefit. Mechanistic analysis further showed that LysE preferentially exports lysine over 5-AVD, establishing it as a flux gatekeeper that critically shapes product selectivity. Overexpression of heterologous NADP⁺-dependent glyceraldehyde-3-phosphate dehydrogenase (GapN) enhanced NADPH supply and improved redox balance, increasing the 5-AVD yield to 0.32 mol mol^−1^ in strain AVD-11. In fed-batch fermentation, AVD-11 reached a maximum productivity of 1.2 g L^−1^ h^−1^ and a final titer exceeding 36 g L^−1^ with > 97% selectivity, while chromosomally integrated *davB* remained genetically stable throughout the process.

**Conclusions:**

This study establishes *C. glutamicum* as a robust and industrially relevant platform for the sustainable production of 5-AVD. By combining rational pathway design, transporter control, and cofactor engineering, we deliver the first high-yield microbial route to this valuable amide and provide a blueprint for expanding the portfolio of lysine-derived monomers accessible through microbial cell factories.

**Supplementary Information:**

The online version contains supplementary material available at 10.1186/s12934-026-02922-1.

## Background

The growing demand for sustainable, bio-based materials has driven increasing interest in renewable platform chemicals derived from amino acids and other primary metabolites. Among these, 5-aminovalerate (5-AVA) has gained considerable attention as a versatile C5 building block for the synthesis of polyamides, specialty chemicals, and high-performance biopolymers [1, 2].

**Fig. 1:**
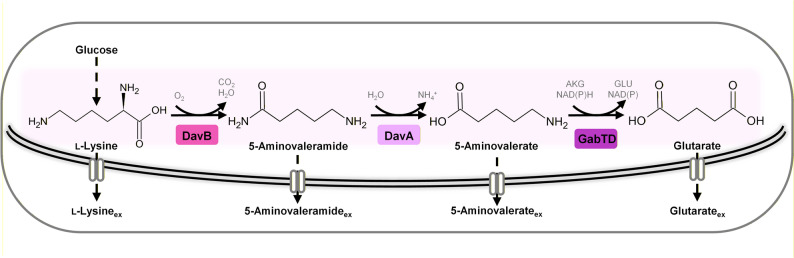
Heterologous metabolic pathway for the synthesis of 5-aminovaleramide, 5-aminovalerate, and glutarate in *C. glutamicum*

In contrast, the structurally related amide 5-aminovaleramide (5-aminopentanamide, 5-AVD) has remained largely unexplored despite its promising industrial potential. In the native l-lysine (hereafter lysine) catabolism of *Pseudomonas putida*, lysine is first converted to 5-AVD by the lysine 2-monooxygenase DavB and subsequently hydrolyzed by the 5-aminovaleramide amidohydrolase DavA to 5-AVA and ammonium [2, 3]. Thus, 5-AVD is the direct precursor of 5-AVA in this pathway (Fig. 1). To our knowledge, a physiologically relevant reverse reaction of DavA has not been reported. Thermodynamic analysis indicates that the hydrolysis of 5-AVD to 5-AVA is energetically favored (ΔG′° = −12.0 ± 4.5 kJ mol^−1^), whereas the reverse reaction can likely be neglected under physiological conditions [4]. Despite its potential, systematic research on 5-AVD has been very limited, mainly because of its poor commercial availability and the historical absence of analytical standards [5]. Consequently, earlier studies could not experimentally verify its accumulation or properties. Only recently have commercial standards apparently become available, although they remain expensive (about 1000 USD per gram). In our work, an unknown side product detected in an imbalanced 5-AVA-producing strain raised the hypothesis that it could be 5-AVD. After acquiring such a standard, we were able to confirm this identity, which subsequently motivated the development of a dedicated biosynthetic route.

From an application perspective, 5-AVD is an attractive bifunctional monomer containing both amino and amide groups. It can be cyclized to δ-valerolactam, a key intermediate for the synthesis of polyamide-5 (PA5) and various co-polyamides with desirable mechanical and thermal properties [6]. Beyond polyamides, 5-AVD may also serve as a precursor for high-performance polyurethanes, biodegradable polymers, and specialty solvents, making it a valuable building block for the emerging bio-based polymer industry [7]. At present, 5-AVD production remains confined to chemical synthesis, relying on multi-step petrochemical routes such as ammonolysis of glutaric derivatives [8], dibromopentane conversion, or butadiene hydrocyanation [9]. These methods employ hazardous reagents (e.g., HCN, nitriles), require high temperatures and strong catalysts, and generate considerable by-products resulting in low selectivity, limited substrate availability, and high production costs. Their dependence on fossil-derived feedstocks and poor environmental compatibility further constrain scalability. Consequently, the development of a biotechnological process using renewable substrates such as glucose or lysine represents a sustainable, safe, and economically attractive alternative for 5-AVD manufacturing.

To date, no targeted biosynthetic route for 5-AVD has been reported. However, microbial production of related compounds such as 5-AVA and glutarate (GTA) has been extensively explored through both de novo metabolic engineering and whole-cell biotransformation of lysine. In many of these approaches, the lysine catabolism enzymes DavB and DavA from *P. putida* were employed, implying transient formation of 5-AVD as an intermediate that is normally hydrolyzed immediately to 5-AVA [2, 5, 10, 11]. In a two-enzyme coupled system developed for synthesizing 5-AVA from lysine, accumulation of 5-AVD was observed when DavA was omitted [10], demonstrating that 5-AVD can accumulate when the hydrolytic step is blocked. Previous work with metabolically engineered *Corynebacterium glutamicum* reported high 5-AVA titers of up to 51.8 g L^−1^, together with residual lysine and GTA as by-products, whereas 5-AVD was not detected or quantified in these studies [1, 2, 12]. Other studies focusing on de novo 5-AVA synthesis in *Escherichia coli* also highlighted the potential of 5-AVD as a valuable product, though it was not further investigated due to the lack of commercially available standards [5]. Whole-cell biocatalysis strategies converting lysine in *E. coli* have achieved titers exceeding 240 g L^−1^ [13]. These studies collectively demonstrate the potential of microbial systems for valorizing lysine into C5 platform chemicals and highlight 5-AVD as a key yet unexplored intermediate and product candidate.

The present work establishes metabolically engineered *C. glutamicum* as a microbial cell factory for the selective and high-yield production of 5-AVD. By combining pathway optimization, transporter screening, and redox balance control, we achieved unprecedented titers of this amide monomer. The resulting process represents a sustainable alternative to conventional chemical synthesis and lays the foundation for the development of biobased polyamide precursors. To achieve this, we introduced and optimized expression of the *davB* gene in the lysine–overproducing *C. glutamicum* LYS-12 strain [14], enabling the direct conversion of lysine into 5-AVD and resulting in detectable secretion of the product into the culture supernatant.

To further enhance productivity, we implemented successive rounds of metabolic engineering, culminating in the construction of *C. glutamicum* AVD-11. This genome-based strain exhibited markedly improved 5-AVD yields and productivities in both batch and fed-batch fermentations, achieving the first reported de novo microbial production of this promising bio-based monomer.

## Results

### Imbalanced expression of *davBA* in *C. glutamicum* causes secretion of 5-aminovaleramide (5-AVD)

**Fig. 2:**
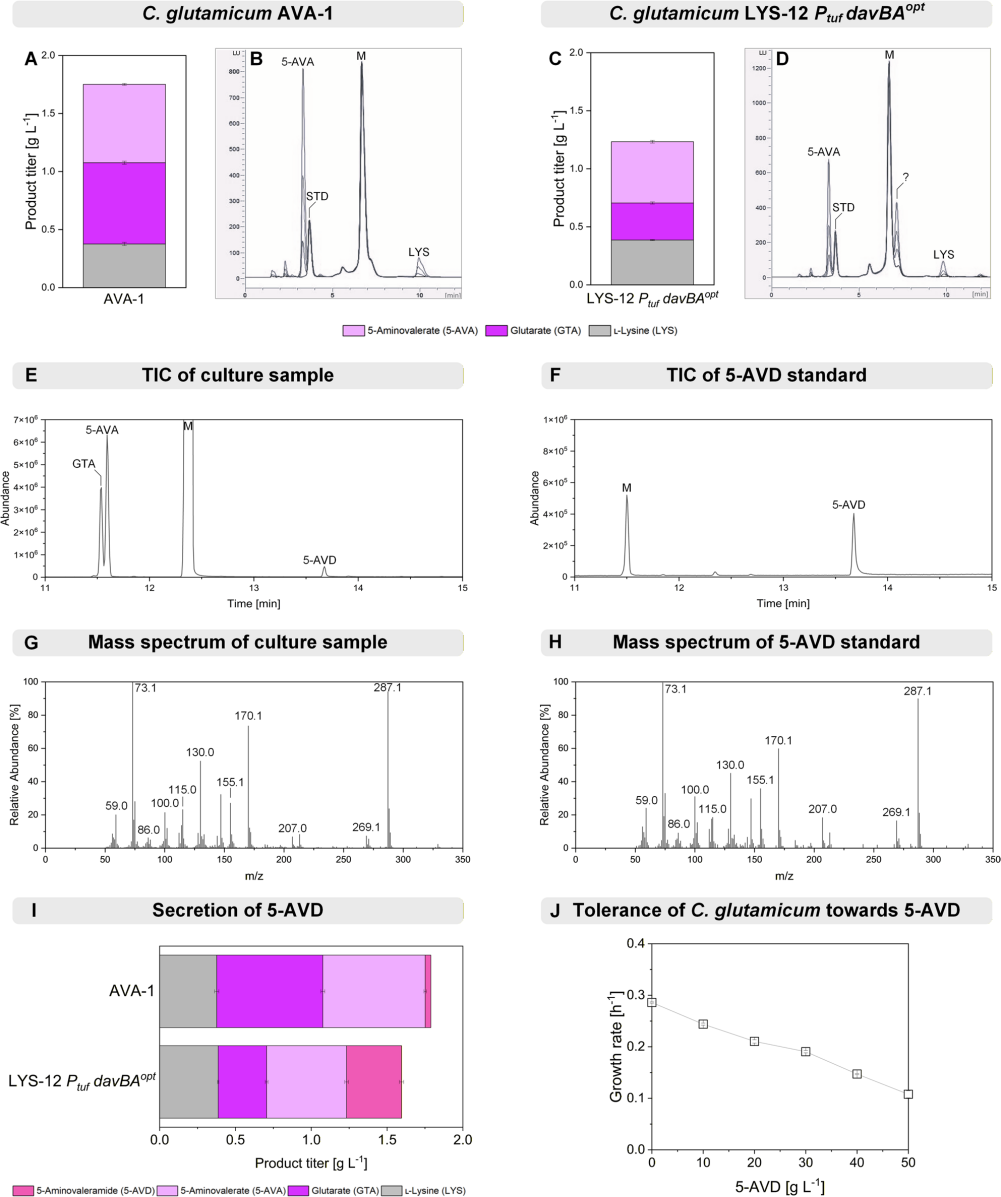
Identification of 5-aminovaleramide as a novel by-product in *C. glutamicum* LYS-12 *P*_*tuf*_ *davBA*^*opt*^. HPLC analysis revealed that codon-optimized *davBA* expression from *P. putida* led to reduced product titers (**A**, **C**) and formation of a novel by-product (**B**, **D**), identified as 5-aminovaleramide (5-AVD) by comparison with a commercial standard (**E**–**H**). The mass spectra correspond to the 5-AVD peak, eluting after 13.88 min. *C. glutamicum* LYS-12 *P*_*tuf*_ *davBA*^*opt*^ accumulated nearly tenfold more 5-AVD than the strain with native *davBA* (**I**). Owing to its tolerance to high levels of 5-AVD (**J**), *C. glutamicum* represents a promising host for targeted production

In previous work, *C. glutamicum* was engineered to overproduce 5-AVA and GTA by extending the lysine biosynthetic pathway with heterologous *davBA* expression [1, 2]. The lysine overproducer *C. glutamicum* LYS-12 was selected as the chassis strain due to its high capacity for lysine accumulation [14]. Two mutants, constructed in the previous study, were grown in minimal glucose medium: LYS-12 *P*_*tuf*_ *davBA*^*nat*^ (AVA-1), expressing *davBA* with native codon usage, and LYS-12 *P*_*tuf*_ *davBA*^*opt*^ (previously named AVA-1 *P*_*tuf*_ *davBA*^*opt*^) carrying codon-optimized variants. As observed before, both strains secreted mixtures of 5-AVA and GTA, alongside residual lysine (Fig. 2A, C).

Interestingly, the strain harboring codon-optimized *davBA* secreted markedly less GTA and 25% less 5-AVA, but accumulated substantial amounts of an unknown by-product. This compound, detected by fluorescence analysis after *o*-phthaldialdehyde derivatization, was presumed to contain an amino group. GC-MS analysis revealed a distinct peak in culture GTA and 5-AVA profiles (Fig. 2D). To identify the metabolite, LYS-12 *P*_*tuf*_ *davBA*^*opt*^ was cultivated in tracer medium containing 99% ^15^N-labeled ammonium sulfate. Several mass fragments displayed a + 2 Da shift, indicating two nitrogen atoms and suggesting the compound to be 5-AVD, the amide intermediate in the 5-AVA/GTA pathway (Additional File 1, Fig. S1). Identity was confirmed by comparison with a commercial standard, showing identical retention time and mass fragmentation (Fig. 2E–H).

Notably, 5-AVD secretion was not observed in previous work and is documented here for the first time. This accumulation likely resulted from an imbalance in DavB and DavA activities caused by codon-optimization. In our earlier study [2], the native codon variant (AVA-1) displayed balanced enzyme activities, whereas the codon-optimized strain (AVA-1 *P*_*tuf*_ *davBA*^*opt*^) exhibited a 97% reduction in DavA and a 40% reduction in DavB activity, shifting the ratio between both enzymes and promoting accumulation of the intermediate 5-AVD. Consistent with this observation, earlier studies reported that codon-optimization of *davBA* genes produced divergent outcomes, ranging from reduced 5-AVA yields and cell viability [15] to enhanced production when combined with additional pathway modifications [16].

Because reuptake could counteract product secretion, we tested whether *C. glutamicum* LYS-12 can import 5-AVD by supplying 1 mM (116 mg L^−1^) externally. After 20 h, 5-AVD remained unconsumed. This indicates the absence of a native 5-AVD import system, consistent with the inability of *C. glutamicum* to catabolize 5-AVD as sole carbon source (Additional File 1, Fig. S2).

Tolerance assays further revealed the robustness of the host strain. To account for the high cost of commercial 5-AVD, agar-based tests were performed in a miniaturized 48-well plate format. *C. glutamicum* LYS-12 grew in the presence of up to 50 g L^−1^ 5-AVD, the highest concentration tested (Additional File 1, Fig. S3). To obtain a quantitative assessment, tolerance experiments were conducted using a miniaturized liquid culture setup. *C. glutamicum* maintained growth up to 50 g L^−1^ 5-AVD, reaching approximately 40% of the maximum growth rate of the non-supplemented control (Fig. 2J, Additional File 1, Fig. S3). Thus, *C. glutamicum* exhibits high intrinsic tolerance to 5-AVD, comparable to its previously reported tolerance toward 5-AVA [2] and GTA [17], both of which have been produced at high titers.

### Plasmid-based expression of lysine 2-monooxygenase in *C. glutamicum* LYS-12 enables the controlled production of 5-aminovaleramide (5-AVD)

To enable tailored production of 5-AVD, the gene *davB*, encoding lysine 2-monooxygenase, was expressed heterologously in *C. glutamicum* LYS-12. To assess the impact of codon usage on expression, two constructs were generated: one harboring the native *davB* sequence and the other a codon-optimized variant adapted to the codon usage preferences of *C. glutamicum*. Both gene variants were placed under the control of the constitutive *tuf* promoter and cloned into the self-replicating plasmid *pClik 5a*. Transformation of *C. glutamicum* LYS-12 yielded strains AVD-1A (native *davB*) and AVD-1B (codon-optimized *davB*).

**Fig. 3:**
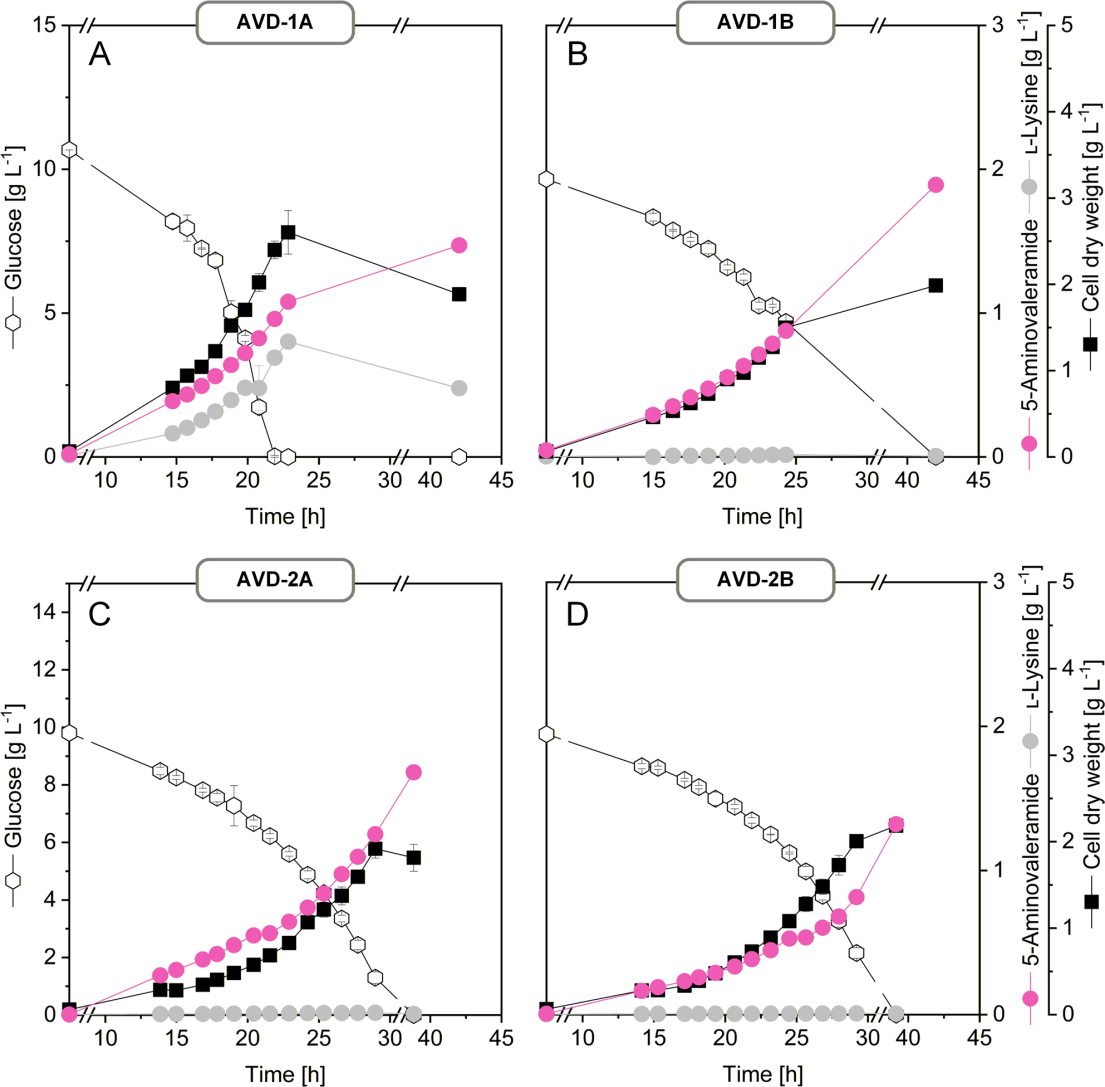
Performance of first-generation 5-aminovaleramide-producing *C. glutamicum* strains. Initial strains were constructed by episomal expression of native (AVD-1A) (**A**) or codon-optimized (AVD-1B) (**B**) *davB* from *P. putida* under the *tuf* promoter in *C. glutamicum* LYS-12. The corresponding *P*_*tuf*_-*davB* modules were subsequently integrated into the *bioD* locus, yielding AVD-2A (**C**) and AVD-2B (**D**). Strains were cultivated in glucose minimal medium at 30 °C in baffled shake flasks. All strains selectively accumulated 5-AVD, while neither 5-aminovalerate nor glutarate was detected by HPLC (< 0.05 g L^−1^), consistent with the absence of *davA* and negligible spontaneous hydrolysis of 5-AVD. Data represent mean ± standard error from three biological replicates

Shake flask cultivations in glucose-based minimal medium demonstrated that both strains redirected carbon flux efficiently toward 5-AVD, reaching product yields above 0.2 mol mol^−1^ glucose. Strain AVD-1B achieved nearly complete selectivity for 5-AVD (99%) but suffered from impaired growth, whereas AVD-1A maintained higher fitness but secreted a mixed product spectrum of 5-AVD (58%) and residual lysine (42%) (Table 1; Fig. 3). These results demonstrate that introduction of *davB* enables a strong rerouting of the intracellular lysine flux toward 5-AVD, despite the parental strain’s metabolic background being optimized for high-level lysine accumulation and export [14]. Because the 5-AVD producers harbored *davB* only and lacked *davA*, enzymatic hydrolysis of 5-AVD to 5-AVA could not occur in these strains. Spontaneous hydrolysis was expected to be negligible under the cultivation conditions. Consistent with this, HPLC analysis of culture supernatants from AVD-1A and AVD-1B did not detect 5-AVA or GTA (detection limit < 0.05 g L^−1^), indicating selective accumulation of 5-AVD in *davB*-based producers without measurable downstream conversion.

**Table 1:** Kinetics and stoichiometry of growth and product formation in first generation 5-aminovaleramide-producing *C. glutamicum* strains

	AVA-1	LYS-12 *P*_tuf_ davBA^opt^	AVD-1A	AVD-1B	AVD-2A	AVD-2B
Rates						
µ (h^−1^)	0.21 ± 0.00	0.21 ± 0.00	0.15 ± 0.01	0.12 ± 0.01	0.16 ± 0.01	0.15 ± 0.01
q_GLC_ (mmol g^−1^ h^−1^)	3.9 ± 0.3	3.5 ± 0.1	4.44 ± 0.66	3.29 ± 0.43	3.28 ± 0.28	3.00 ± 0.20
q_5-AVD_ (mmol g^−1^ h^−1^)	0.01 ± 0.00	0.31 ± 0.02	0.53 ± 0.15	0.70 ± 0.05	0.77 ± 0.07	0.40 ± 0.02
q_LYS_ (mmol g^−1^ h^−1^)	0.24 ± 0.02	0.20 ± 0.00	0.38 ± 0.08	0.01 ± 0.00	0.01 ± 0.00	0.00 ± 0.00
q_5-AVA_ (mmol g^−1^ h^−1^)	0.35 ± 0.02	0.34 ± 0.01	n.d.	n.d.	n.d.	n.d.
q_GTA_ (mmol g^−1^ h^−1^)	0.45 ± 0.06	0.18 ± 0.00	n.d.	n.d.	n.d.	n.d.
Yields						
Y_X/GLC_ (g mol^−1^)	52.2 ± 6.3	62.0 ± 2.8	34.8 ± 2.2	37.8 ± 5.0	43.2 ± 0.85	50.2 ± 2.3
Y_5 − AVD/GLC_ (mmol mol^−1^)	3.3 ± 0.6	91.9 ± 5.7	117.6 ± 14.3	215.4 ± 14.7	234.9 ± 3.4	132.9 ± 0.3
Y_LYS/GLC_ (mmol mol^−1^)	72.0 ± 5.4	58.9 ± 1.5	85.5 ± 11.0	2.4 ± 0.3	1.5 ± 0.2	0.7 ± 0.1
Y_5 − AVA/GLC_ (mmol mol^−1^)	91.7 ± 2.3	100.9 ± 1.3	n.d.	n.d.	n.d.	n.d.
Y_GTA/GLC_ (mmol mol^−1^)	138.0 ± 9.1	54.3 ± 1.5	n.d.	n.d.	n.d.	n.d.

The producers were based on expression of native and codon-optimized *davBA* (AVA-1, LYS-12 *P*_*tuf*_ *davBA*^*opt*^) or the corresponding *davB*-encoded monooxygenase alone (AVD-1 A/B, AVD-2A/B) from *P. putida* KT2440 under control of *P*_*tuf*_. The strains were grown in shake flasks on glucose minimal medium at 30 °C. The data comprise rates of growth (µ), substrate consumption and product formation (q), as well as yields (Y), representing mean values and standard errors from three biological replicates

Previous metabolic-engineering studies targeting reactions associated with the lysine pathway have shown that strong pathway redirection can influence intracellular precursor pools and growth-coupled intermediates [18–20], with diaminopimelate potentially limiting growth [21]. Therefore, we quantified intracellular levels to examine whether similar effects occurred in 5-AVD-producing strains. Diaminopimelate serves as both a lysine pathway intermediate and an essential precursor for peptidoglycan synthesis in *C. glutamicum* [21]. Strain AVD-1B, which produced the highest 5-AVD titers, showed notably lower intracellular levels (3.1 ± 0.2 µmol g^−1^) compared with LYS-12 (5.0 ± 0.6 µmol g^−1^) and the moderate producer AVD-1A (5.6 ± 0.1 µmol g^−1^), suggesting that the strong flux redirection toward 5-AVD formation may have partially depleted intermediates required for cell wall synthesis, thereby potentially contributing to the reduced growth rate observed in the high-producing strain AVD-1B.

### Heterologous lysine 2-monooxygenase enables high-yield 5-AVD production via single-copy genomic expression of *davB*

To eliminate the need for plasmid maintenance—which typically requires selection markers such as antibiotics [22] and can impose metabolic burden [23]—*C. glutamicum* was engineered for chromosomal integration of *davB*. This genome-based expression strategy was designed to enable stable, selection-free 5-AVD production while avoiding instability and the resource burden associated with episomal systems. Native and codon-optimized *P*_*tuf*_*-davB* expression cassettes (1,883 bp) were integrated into the *bioD* locus of *C. glutamicum* LYS-12, a preferred genomic site for heterologous gene expression [2]. The resulting strains, AVD-2A (harboring the native *davB*) and AVD-2B (harboring the codon-optimized *davB*), were confirmed by PCR and Sanger sequencing.

In shake flask cultivations, strain AVD-2A achieved a 5-AVD yield of 0.20 mol mol^−1^ glucose, accompanied by a pronounced shift in the product spectrum toward 5-AVD compared to its plasmid-based counterpart. Moreover, AVD-2A exhibited faster growth and a higher specific production rate, consistent with the reduced metabolic burden of chromosomal expression. In contrast, AVD-2B, while also showing improved growth performance, produced lower 5-AVD yields and rates than the plasmid-based strain AVD-1B. This finding suggests that the previously observed superiority of AVD-1B over AVD-1A was not a result of codon optimization but rather a consequence of the growth impairment associated with high-level plasmid-based expression (Table 1; Fig. 3) and partial depletion of growth-coupled intermediates.

### Promoter engineering of *davB* reveals flux bottlenecks and competition for intracellular lysine

**Fig. 4:**
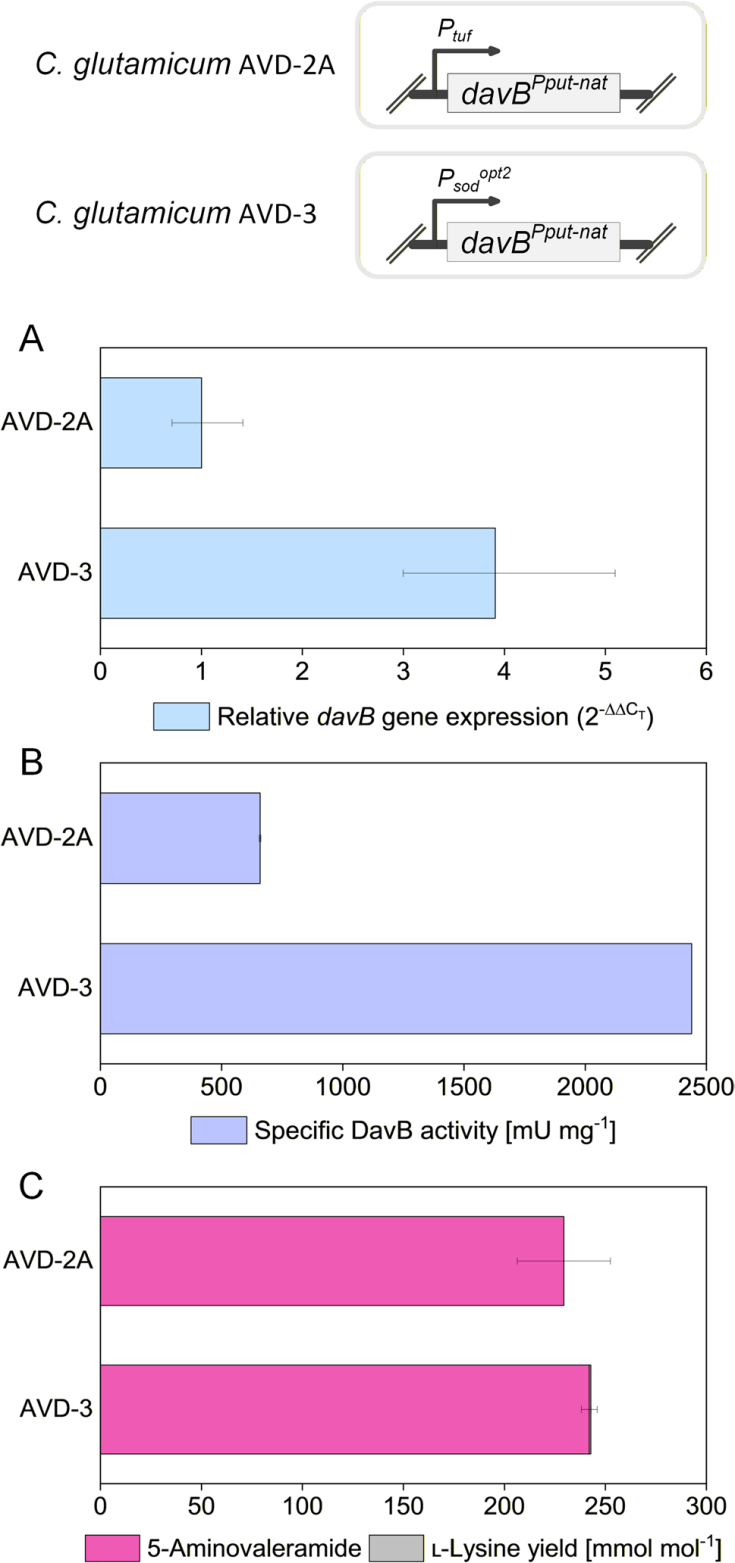
Characterization of lysine 2-monooxygenase from *P. putida* in 5-aminovaleramide overproducing *C. glutamicum* strains. Cell extracts from exponentially growing strains (LYS-12, AVD-2A, AVD-3) were assayed at 30 °C in baffled shake flasks. Reactions were initiated by adding 500 µL extract to 9.5 mL master mix (20 mM lysine, 100 mM phosphate buffer, pH 7.8). Specific activities and product yields are reported as mean ± standard error from three technical replicates

To enhance pathway flux, *davB* expression was increased by replacing the *tuf* promoter with the stronger *sod*^*opt2*^ promoter, a mutagenized variant of the *C. glutamicum sod* promoter [18]. RT-qPCR analysis confirmed that *davB* transcript levels in AVD-3 were 3.8-fold higher than in AVD-2A, demonstrating the expected increase in gene expression resulting from promoter replacement (Fig. 4). Consistent with this, in vitro analysis of lysine 2-monooxygenase activity in cell extracts showed that AVD-3 exhibited a 3.7-fold elevated specific activity (nearly 2500 mU mg^−1^) compared to AVD-2A (670 mU mg^−1^). However, the substantial increase in *davB* expression and enzyme activity translated only into a modest improvement in 5-AVD yield, although production remained fully selective with no detectable lysine by-product (Table 2). These results indicate that, beyond DavB activity , additional bottlenecks constrain pathway flux, which must be addressed to further improve production.

**Table 2:** Kinetics and stoichiometry of growth and product formation in advanced 5-aminovaleramide-producing *C. glutamicum* strains

	AVD-2A	AVD-3	AVD-10	AVD-11
Rates				
µ (h^−1^)	0.17 ± 0.01	0.11 ± 0.04	0.10 ± 0.01	0.12 ± 0.02
q_GLC_ (mmol g^−1^ h^−1^)	3.09 ± 0.05	3.35 ± 0.09	3.10 ± 0.15	3.45 ± 0.24
q_5-AVD_ (mmol g^−1^ h^−1^)	0.72 ± 0.02	0.79 ± 0.12	1.00 ± 0.08	1.09 ± 0.09
q_LYS_ (mmol g^−1^ h^−1^)	0.00 ± 0.00	0.00 ± 0.00	0.01 ± 0.01	0.00 ± 0.00
Yields				
Y_X/GLC_ (g mol^−1^)	56.1 ± 3.4	32.3 ± 11.7	33.2 ± 3.8	35.4 ± 3.6
Y_5 − AVD/GLC_ (mmol mol^−1^)	233.1 ± 4.8	235.6 ± 30.8	320.7 ± 16.9	316.0 ± 4.8
Y_LYS/GLC_ (mmol mol^−1^)	0.8 ± 0.1	0.0 ± 0.0	4.6 ± 2.4	0.0 ± 0.0

The producers were based on the expression of the native *davB* from *P. putida* KT2440 under control of the promoter *P*_*tuf*_ (AVD-2A, AVD-10) and *P*_*sod*_^*opt2*^ (AVD-3, AVD-11), respectively. In addition, the codon-optimized *gapN* module under control of *P*_*tuf*_ is expressed (AVD-10 and AVD-11). All strains were grown in shake flasks on glucose minimal medium at 30 °C. The data comprise rates of growth (µ), substrate consumption and product formation (q), as well as yields (Y), representing mean values and standard errors from three biological replicates

*GLC* glucose, *5-AVD* 5-aminovaleramide, *LYS* lysine, *X* biomass

**Fig. 5:**
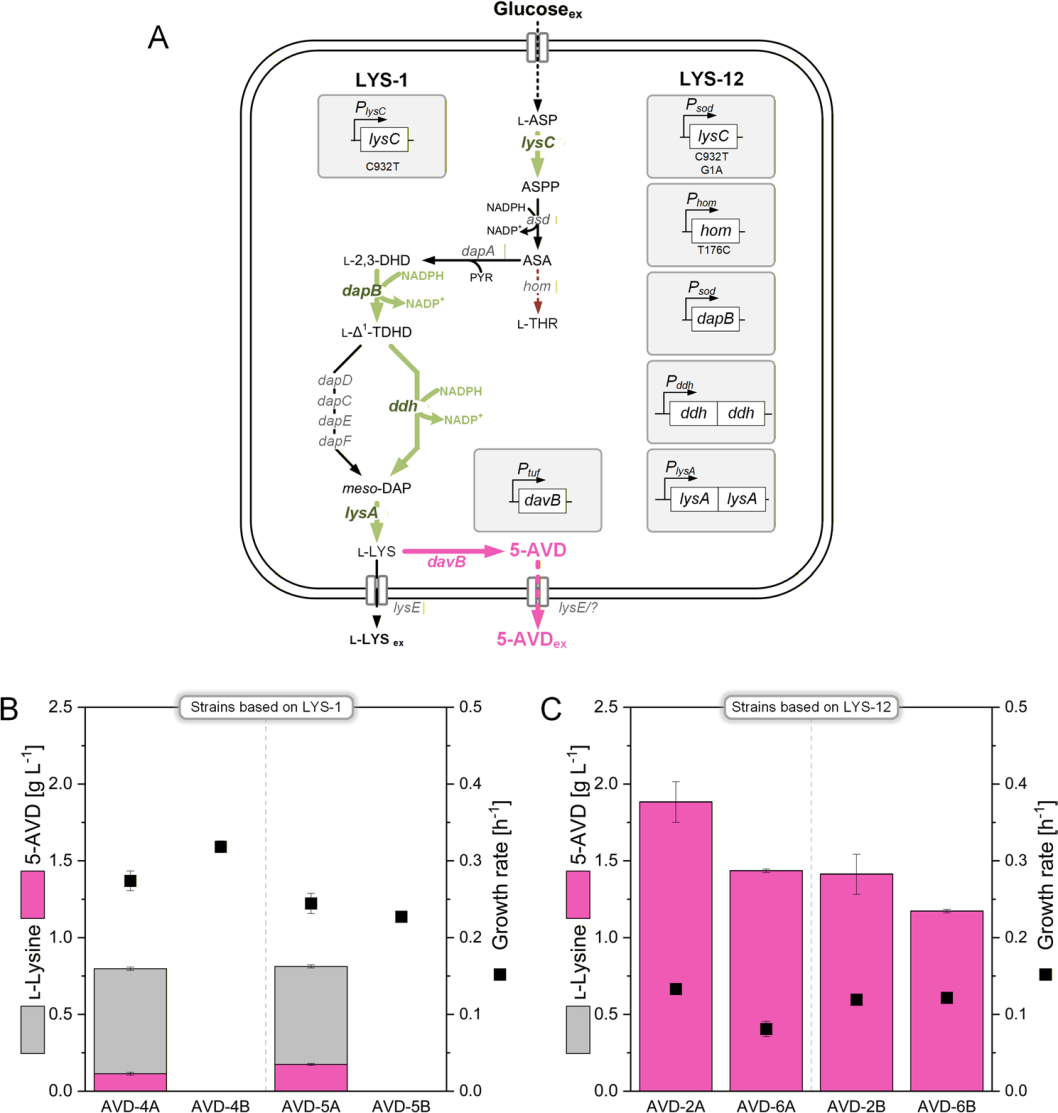
Effect of lysE deletion on 5-aminovaleramide production in C. glutamicum. Strains expressing Ptuf davB either episomally in the basic lysine producer LYS-1 (A) or genomically in the advanced producer LYS-12 (B) were compared with their respective lysE-deficient derivatives. The data represent the yields for lysine and 5-AVD and the specific growth rate. Cultivations were performed in a miniaturized bioreactor system with glucose minimal medium. Data represent mean ± standard error from three biological replicates

To explore the role of intracellular lysine availability, 5-AVD biosynthesis was also established in the basic producer LYS-1, which accumulates significantly less lysine than LYS-12 and harbors only a feedback-resistant aspartokinase as its sole genetic modification [14]. Episomal expression of native and codon-optimized *P*_*tuf*_*-davB* modules in LYS-1 generated strains AVD-4A and AVD-5A, respectively. Both strains secreted only minor amounts of 5-AVD (0.12–0.17 g L^−1^, 1–1.5 mM) but released 0.67 g L^−1^ lysine (4.6 mM) as the dominant product. This preferential secretion of lysine suggests competition between the native exporter LysE and lysine 2-monooxygenase DavB for the intracellular lysine pool. In low-producing strains, limited lysine availability appears to favor export rather than conversion, whereas in high-producing strains, elevated intracellular concentrations enable efficient enzymatic conversion to 5-AVD, thereby promoting its secretion (Fig. 5).

### The lysine exporter LysE contributes to but does not exclusively mediate 5-AVD secretion

In *C. glutamicum*, LysE is the primary lysine exporter, yet it also facilitates the export of other amino acids and related solutes [24]. Due to the structural similarity between lysine and 5-AVD, LysE was hypothesized to participate in 5-AVD export. Given the central role of LysE in lysine export, we examined its impact on 5-AVD formation by deleting the *lysE* gene in the AVD-producing strains. Unlike their LYS-1-based parental strains, which secreted both 5-AVD and lysine, the *lysE*-deficient derivatives AVD-4B and AVD-5B (Δ*lysE*) did not release either compound (Fig. 5). These results indicate that LysE is essential for 5-AVD secretion in low-level producers, and that efficient export is critical to maintain pathway activity.

To exclude confounding effects from the reduced lysine biosynthesis capacity of LYS-1 compared to LYS-12, *lysE* deletions were also introduced into higher-producing strains AVD-2A and AVD-2B, generating AVD-6A and AVD-6B. Unlike earlier reports of difficulties in deleting *lysE* in pipecolic acid producers [18], the deletion proceeded without complications. Interestingly, both AVD-6A and AVD-6B still secreted substantial amounts of 5-AVD, albeit at lower levels than their parental strains, while lysine secretion was abolished (Fig. 5, Additional File 1, Table S1). This suggests that although LysE contributes to 5-AVD export, additional transport systems must be involved. Unexpectedly, no intracellular accumulation of 5-AVD was observed in *lysE*-deficient strains (Additional File 1, Table S2). Instead, biosynthesis of both lysine and 5-AVD appeared downregulated, pointing to an indirect regulatory effect triggered by impaired export rather than a strict transport limitation.

### Evaluation of heterologous GABA permeases provides insights into 5-AVD export specificity

To explore alternative strategies for enhancing 5-AVD secretion, we investigated the potential contribution of γ-aminobutyric acid (GABA) permeases. This approach was motivated by previous findings where heterologous expression of the GABA permease GabP from *P. putida* improved export of the structurally related compound 5-aminovalerate (5-AVA) in *C. glutamicum* [2]. To assess whether GabP could also facilitate 5-AVD transport, GabP-III (PP2911) from *P. putida*, expressed under the *tuf* promoter (1,592 bp), was integrated into the *gabTDP* locus of strains AVD-2A and AVD-3. The resulting strains, AVD-7A (AVD-2A *P*_*tuf*_ *gabP*^*Pput*^) and AVD-7B (AVD-3 *P*_*tuf*_ *gabP*^*Pput*^) showed reduced growth and slightly decreased 5-AVD yields, indicating that GabP-III does not enhance 5-AVD secretion.

To broaden the analysis, three additional *gabP* genes encoding GABA permeases were selected: one from *Escherichia coli* (2,806 bp) [25] and two from *Mycobacterium smegmatis* (2,828 bp and 3,233 bp) [26]. To avoid the fitness burden of plasmid-based overexpression, each gene was genomically integrated into AVD-2A or AVD-2B, generating strains AVD-8A (AVD-2A *P*_*tuf*_ *gabP*^*Msme1*^), AVD-8B (AVD-2A *P*_*tuf*_ *gabP*^*Msme2*^), AVD-9A (AVD-2B *P*_*tuf*_ *gabP*^*Msme1*^), and AVD-9B (AVD-2B *P*_*tuf*_ *gabP*^*Ecol*^). Correct integration was verified by PCR and sequencing. None of these engineered strains displayed improved 5-AVD secretion. Most produced levels comparable to the parental strains, whereas AVD-9B (*gabP*^*Ecol*^) secreted less 5-AVD while releasing increased amounts of lysine.

To confirm that the lack of improved 5-AVD secretion was not due to insufficient transporter expression, we quantified mRNA levels of one representative *gabP* gene from each species (*M. smegmatis*, *E. coli*, *P. putida*). All three genes were highly expressed in the corresponding engineered strains, as demonstrated by qRT-PCR using *sigA* as reference (Supplementary Fig. S4). These results demonstrate that the heterologous permeases were transcriptionally active. Together with previous studies showing correct membrane insertion and activity of GABA permeases heterologously expressed in *C. glutamicum* [2], these findings support that the absence of a secretion effect originates from substrate specificity rather than failed expression.

These findings indicate that the tested heterologous GABA permeases do not facilitate 5-AVD export in *C. glutamicum* (Additional File 1, Fig. S4). In fact, 5-AVD transport relies on different or more specific mechanisms, providing a useful basis for future efforts to identify and engineer dedicated 5-AVD exporters.

### Redox balancing via GapN enhances NADPH supply and drives high-yield 5-AVD production

The biosynthesis of 5-AVD is tightly coupled to cellular redox balance. In particular, the formation of its precursor lysine requires 4 mol NADPH per mol, underscoring the importance of cofactor supply for efficient flux through the pathway [27–29]. In *C. glutamicum*, increasing NADPH availability has long been a cornerstone of metabolic engineering for improved lysine yields. Accordingly, the chassis strain *C. glutamicum* LYS-12 was pre-engineered with pentose phosphate (PP) pathway modifications that enhanced NADPH regeneration [14]. These background modifications were preserved in all 5-AVD producers and, likely, contributed to the observed product yields.

As limited NADPH supply appeared to constrain 5-AVD formation, we enhanced redox regeneration by introducing the NADP⁺-dependent GapN from *S. mutans*. GapN directly links glycolytic flux from glyceraldehyde-3-phosphate to 3-phosphoglycerate while generating NADPH [30, 31]. Building on the genome-integrated producers AVD-2A and AVD-3, we constructed strains AVD-10 and AVD-11. In AVD-10, *gapN* was expressed under the *tuf* promoter alongside *davB* (native sequence), while in AVD-11, *gapN* was driven by *tuf* and *davB* by the stronger *sod*^*opt2*^ promoter. Genomic modifications were confirmed by PCR and sequencing.

**Fig. 6:**
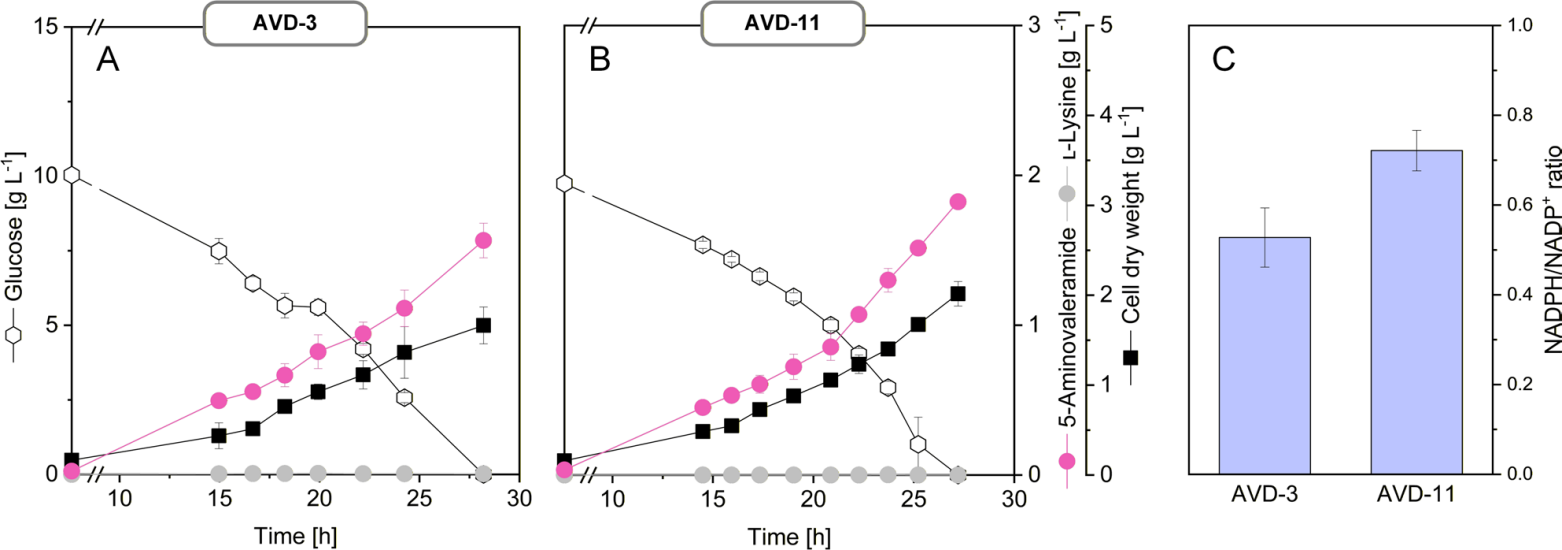
Production performance and intracellular NADPH/NADP⁺ ratio of *C. glutamicum* strains. **A** AVD-3 and **B** AVD-11 expressed native *davB* from *P. putida* KT2440 under the *sod*^*opt2*^ promoter, with AVD-11 additionally carrying the *P*_*tuf*_ *gapN* module. Production performance was analyzed in shake flask cultivations at 30 °C with glucose minimal medium. **C** Intracellular NADPH/NADP⁺ ratios were determined from cultures harvested in mid-exponential phase. Data represent mean ± standard error from duplicates (NADPH/NADP⁺ ratio) and triplicates (cultivation profiles)

When cultivated in glucose-based minimal medium, both GapN-expressing strains demonstrated markedly improved performance (Fig. 6). AVD-10 achieved a 5-AVD yield of 0.32 mol mol^−1^ glucose, representing a 40% increase compared to the parental strain, alongside a 39% improvement in specific productivity. AVD-11 reached a similarly high yield but at an even higher production rate and, notably, secreted 5-AVD with full selectivity, eliminating lysine by-product formation (Table 2). Measurements of intracellular redox state confirmed the mechanistic basis of these improvements: while AVD-3 exhibited a relatively low NADPH/NADP⁺ ratio (0.53 ± 0.07), AVD-11 displayed a significantly elevated ratio (0.72 ± 0.05), consistent with enhanced NADPH regeneration via GapN (Fig. 6). These results demonstrate that fine-tuning the intracellular redox balance is a powerful strategy for increasing both the rate and yield of 5-AVD production.

### Export engineering via LysE overexpression alters product selectivity

Since redox optimization successfully increased the metabolic driving force for 5-AVD formation, we next investigated whether further improvements could be achieved by enhancing product export. To this end, *lysE* was placed under control of the strong constitutive *sod* promoter, yielding strain AVD-12. Unexpectedly, LysE overexpression caused a shift in product distribution: approximately 40% of the total product was secreted as lysine during the early phase of cultivation, reducing the selectivity for 5-AVD. Consistent with observations in the *lysE*-deleted LYS-1-based producers, where reduced intracellular lysine apparently limited 5-AVD secretion, amplification of LysE likely lowered cytosolic lysine levels, favoring direct lysine export over its enzymatic conversion to 5-AVD. Interestingly, this effect was transient—after about half of the available glucose had been consumed, lysine secretion declined and 5-AVD became the exclusive product (Additional File 1, Table S3). These findings suggest that while redox balancing via GapN ensures efficient flux toward 5-AVD, export engineering through LysE overexpression influences the timing and selectivity of product release, pointing to a complex interplay between redox state, metabolic flux, and transport capacity.

### Distinct central-carbon flux regimes shape lysine and 5-AVD production

To obtain a systems-level view of how increasing *davB* expression and redox engineering via *gapN* expression reshaped carbon fluxes, we performed ^13^C metabolic flux analysis for the two 5-AVD producers AVD-3 and AVD-11. Fluxes were compared to the parent lysine producer LYS-12, for which a high-resolution flux map was available from previous work [14].

**Fig. 7:**
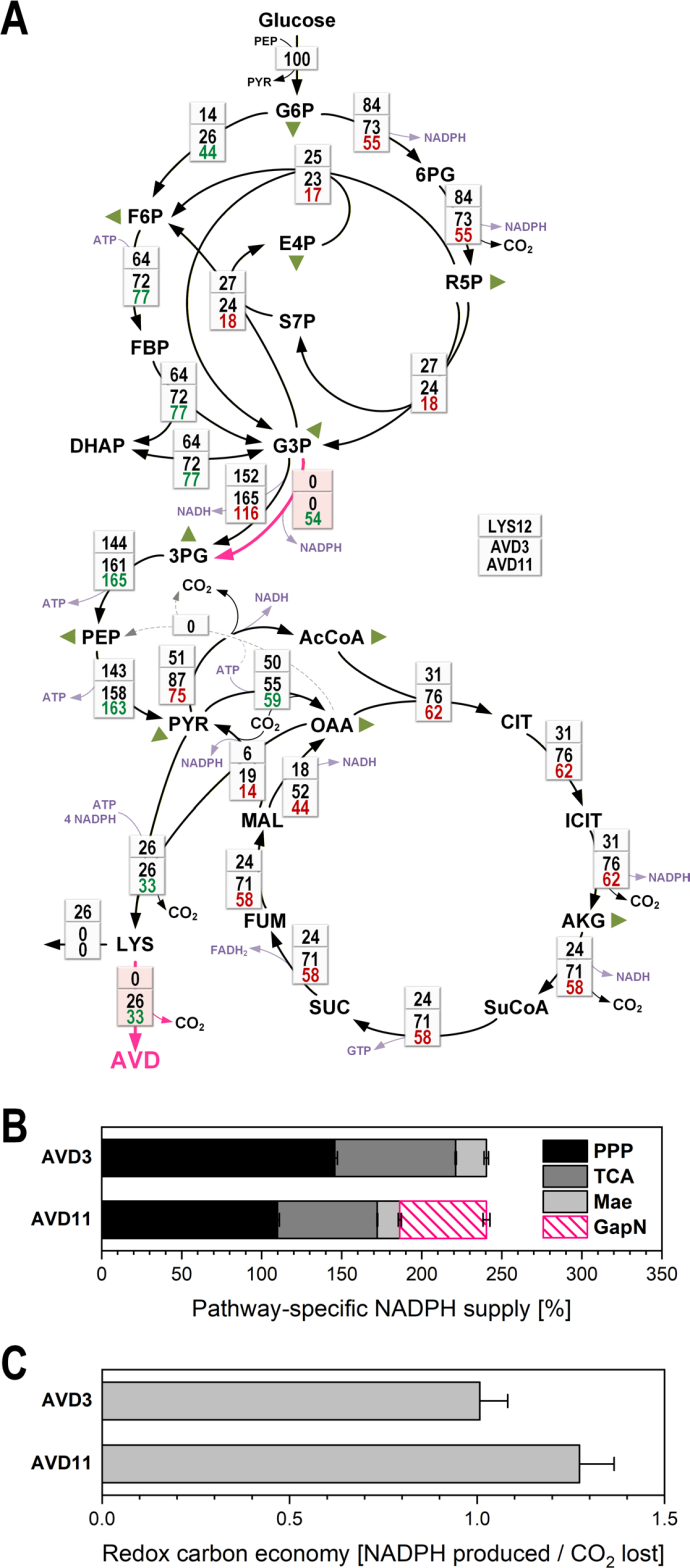
Intracellular carbon fluxes of 5-AVD producing *C. glutamicum* AVD-3 and AVD-11, determined by ^13^C metabolic flux analysis. **A** Relative intracellular flux distribution. All fluxes are expressed as molar percentages of the respective mean specific glucose uptake rates (q_Glc_ = 3.4 mmol g^−1^ h^−1^ for AVD-3 and q_Glc_ = 3.5 mmol g^−1^ h^−1^ for AVD-11), each set to 100%. Fluxes contributing to biomass formation are indicated by green triangles. Flux data of the parent lysine producer LYS-12 are shown for comparison [14]. **B** Pathway-specific contributions to NADPH supply (%) in AVD-3 and AVD-11. **C** Carbon economy of NADPH generation, expressed as the CO_2_ released per NADPH formed (flux ratio, %). Values were calculated from the estimated fluxes using the following stoichiometries: oxidative PP pathway (glucose 6-phosphate dehydrogenase, 6-phosphogluconate dehydrogenase), 2 NADPH per CO_2_ released; GapN, 1 NADPH without CO_2_ release; TCA cycle (isocitrate dehydrogenase), 1 NADPH per 2 CO_2_ released; malic enzyme, 1 NADPH per CO_2_ released. For AVD-11, the individual GAPDH and GapN fluxes were inferred by balancing total NADPH-supplying fluxes, as detailed in “Materials and methods” section

Despite showing similar overall product yields—and thus comparable demands for precursor supply and NADPH—the three strains exhibited markedly different flux distributions across the EMP and PP pathways, and the TCA cycle (Fig. 7A). In LYS-12, glucose catabolism was characterized by a strong dependence on the oxidative PP pathway, consistent with its high NADPH demand for lysine biosynthesis. In contrast, both 5-AVD producers shifted a substantially greater fraction of carbon through the EMP pathway and less flux into the PP pathway, a trend most pronounced in AVD-11. Introduction of GapN in AVD-11 caused a substantial part of the glyceraldehyde-3-phosphate flux (53.8%) to be oxidized via the NADP⁺-dependent bypass, while the native NADH-dependent GAP dehydrogenase flux decreased. Thus, GapN not only provided additional NADPH but also redistributed carbon at the G6P node, enabling cells to maintain NADPH availability even at lower oxidative PP pathway flux.

A second major difference emerged at the level of the TCA cycle. Both AVD-3 and AVD-11 showed substantially higher fluxes through pyruvate dehydrogenase and the TCA cycle reactions than LYS-12. This increase was consistent with the lower biomass yields of the 5-AVD strains: with less carbon entering biosynthesis, more carbon remained available for oxidation in the TCA cycle, resulting in elevated CO₂ formation. This behavior resembled metabolic responses observed when *C. glutamicum* grows on carbon sources that limit biomass yield, where excess carbon is preferentially dissipated through oxidative metabolism [28, 32, 33]. In line with this, both 5-AVD producers exhibited a more pronounced back-flux from malate to pyruvate via malic enzyme, indicating that carbon refluxed out of the TCA cycle, likely because downstream sinks—biomass formation and 5-AVD secretion—could not fully accommodate the incoming flux. The increased malic enzyme flux added to the distinct regime in redox supply. Together, these data demonstrate that while the lysine and 5-AVD producers provided the same overall performance in terms of precursor and redox supply for their respective products, they relied on drastically different flux regimes to achieve this (Fig. 7A).

### Fed-batch benchmarking demonstrates robust and high-level 5-AVD production with excellent selectivity

**Fig. 8:**
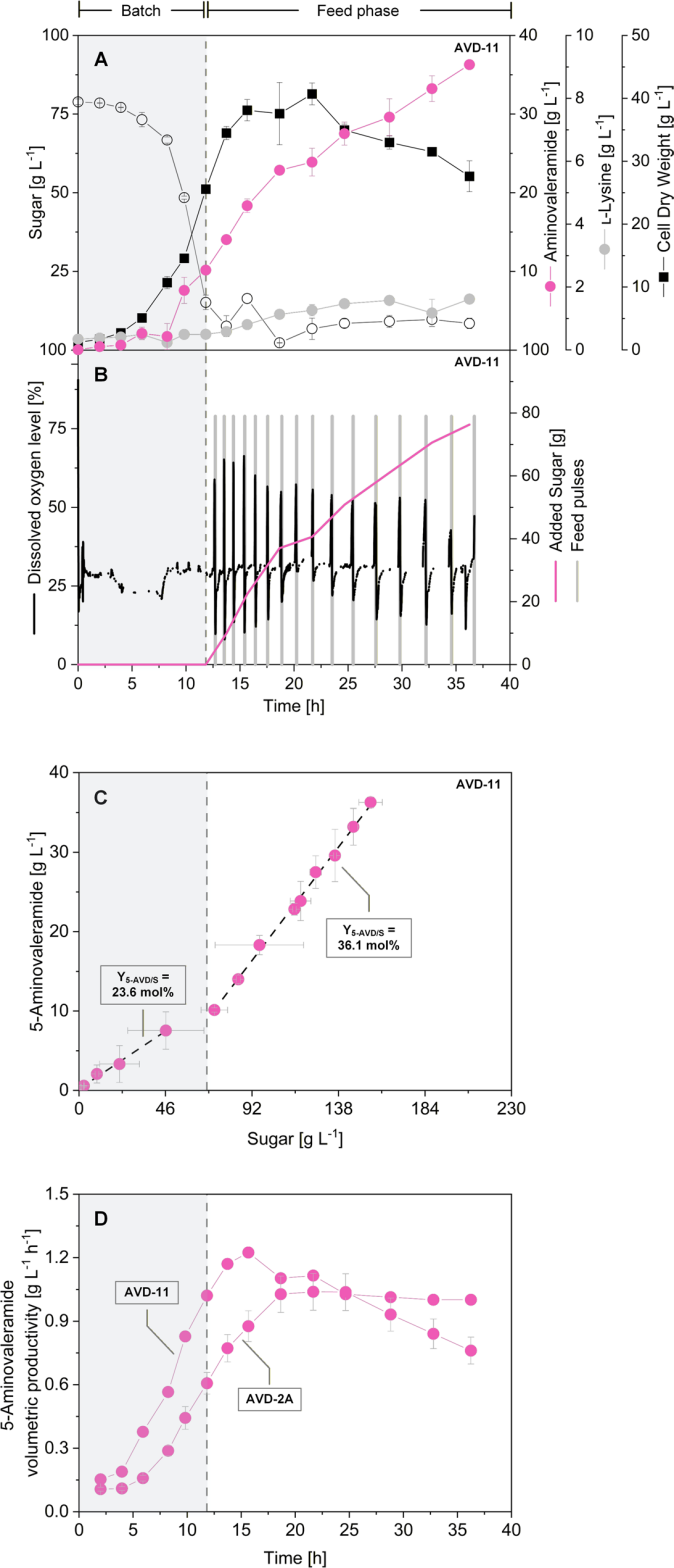
Fed-batch fermentation of *C. glutamicum* AVD-11. **A** Cultivation profile, **B** automated feed addition triggered by dissolved oxygen spikes, and **C** 5-aminovaleramide yield in different process phases. Fermentation was carried out at 30 °C in sucrose–molasses medium. Total sugar represents sucrose, glucose, and fructose. After depletion of the initial substrate, concentrated feed pulses were automatically added when dissolved oxygen rose above 40%. **D** The volumetric productivity of both strains was highest during the feed phase, with a maximal productivity of 1.2 g L^−1^ h^−1^ for strain AVD-11. Data represent mean ± deviation from two biological replicates

To evaluate the industrial potential of the engineered 5-AVD producers, the baseline single-copy *davB* producer AVD-2A (Additional file 1, Fig. S5) and the best-performing producer AVD-11 (Fig. 8) were compared under fed-batch conditions. Cultivations were carried out in a sucrose–molasses medium under conditions relevant for large-scale operation. During the initial batch phase, both strains grew exponentially with identical specific growth rates of 0.28 h^−1^, demonstrating robust utilization of the high starting sugar concentration (80 g L^−1^). 5-AVD secretion began immediately, with batch-phase yields of 19 mol% for AVD-2A and 24 mol% for AVD-11. After 12 h, the initial sugar was depleted, triggering automated feeding controlled by the dissolved oxygen (DO) signal. Pulses of 10 mL of concentrated feed were supplied as required, allowing autonomous process operation without external intervention.

While biomass formation plateaued after 20 h, 5-AVD titers continued to increase throughout the feed phase. Final concentrations reached 27.6 ± 1.7 g L^−1^ for AVD-2A and 36.3 ± 0.5 g L^−1^ for AVD-11 after 36 h. During feeding, the overall yield rose to 36 mol% for both strains. Given the complexity of the molasses-based medium, a slight overestimation of carbon yields cannot be excluded. Minor amounts of lysine were also detected, with final titers of 0.7 g L^−1^ for AVD-2A and 1.6 g L^−1^ for AVD-11, part of which originated from the initial batch medium (0.3 g L^−1^). This corresponds to a selectivity exceeding 97% in both cases. The redox-optimized strain AVD-11 exhibited superior process performance, reaching a maximum space–time yield of 1.2 g L^−1^ h^−1^ during the feed phase and an overall process average of 0.8 g L^−1^ h^−1^. These results highlight the robustness of the engineered strains in complex feedstocks and demonstrate that redox balancing via GapN is directly translatable into industrially relevant process improvements.

To confirm that the strong 5-AVD production phenotype observed in fed-batch was not affected by genetic instability, we verified the integrity of the chromosomally integrated *davB* expression cassette in both AVD-2A and AVD-11 before and after fermentation (Additional file 1, Fig. S6.). PCR analysis targeted the *bioD* integration locus, generating an expected amplicon of 3835 bp in AVD-2A and 3827 bp in AVD-11 carrying the correct integration. Samples were taken at the start of the fermentation (inoculum after preculture) and at the end of the fed-batch fermentation (36 h). Gel electrophoresis showed identical bands at both time points for both strains in all replicates, demonstrating that the *davB* insertion remained intact throughout the entire process.

## Discussion

### Pathway optimization and redox balancing enable efficient 5-AVD biosynthesis

This study demonstrates that *C. glutamicum* can be systematically engineered for the selective production of 5-AVD. Expression of *davB* alone was sufficient to reroute the intracellular lysine pool toward 5-AVD, highlighting the strong driving force of this reaction step. Chromosomal integration of *davB* further stabilized production and reduced the metabolic burden associated with plasmid-based expression [22]. Consistent with these results, RT-qPCR analysis of *P*_*tuf*_- and *P*_*sod*_^*opt2*^-driven *davB* expression confirmed that promoter engineering substantially increased *davB* transcript abundance and enzyme activity, yet the resulting strains did not show proportional increases in 5-AVD formation. This indicates that the major constraints arise elsewhere—most likely linked to precursor availability, redox supply, or transport capacity—rather than from insufficient *davB* expression itself.

Because 5-AVD biosynthesis is highly NADPH-dependent, redox metabolism emerged as a key control point. Introduction of the NADP⁺-dependent glyceraldehyde-3-phosphate dehydrogenase GapN provided an additional carbon-neutral NADPH source at the triose-phosphate node, strengthening cofactor supply and enabling high-yield 5-AVD production. This strategy is particularly useful as it generates NADPH without carbon loss [30]. In particular, strain AVD-11 combined redox optimization with strong *davB* expression, leading to a yield of 0.32 mol mol^−1^ glucose and complete selectivity for 5-AVD. The clear correlation between intracellular NADPH/NADP⁺ ratios and product yields underscores the importance of cofactor engineering for NADPH-intensive pathways [28, 29, 33]. Systematic time-resolved tuning of *davB* expression and activity could help to overcome the remaining flux bottlenecks even under strong constitutive promoter control. In addition, it appears interesting to search for alternative lysine monooxygenases with superior kinetics or stability [34] to further enhance conversion efficiency.

Notably, the process data highlight that AVD-11 outperformed the baseline strain AVD-2A in two distinct process phases (Fig. 8D). During the batch phase, AVD-11 reached both higher 5-AVD yields and increased volumetric productivities, even though biomass formation was still ongoing. This indicates that in AVD-11, NADPH supply was sufficiently strengthened to simultaneously support both growth and 5-AVD biosynthesis, whereas in AVD-2A these two processes appear to compete for redox equivalents. Thus, redox balancing not only increased the metabolic driving force but also alleviated a critical trade-off between biomass generation and product formation.

In the late process phase, the performance gap widened further (Fig. 8D). While AVD-2A gradually phased out, AVD-11 continued to secrete 5-AVD at high rates. This divergence suggests that the improved redox balance in AVD-11 provides metabolic robustness under nutrient-limited or stress conditions, allowing the strain to sustain high-level fluxes even when growth slows. The persistence of volumetric productivity in AVD-11 contrasts with the stagnation observed in AVD-2A, underscoring that the engineered redox supply was a key determinant not only of peak yields but also of long-term process productivity. Further improvements may be possible by engineering global redox cofactor networks. Strategies could include dynamic cofactor balancing through synthetic regulatory circuits [35] or the exploration of alternative NADPH regeneration pathways, such as membrane-bound transhydrogenase [36].

### GapN reconfigures central carbon metabolism and improves the carbon efficiency of NADPH supply

The ^13^C-flux data revealed that *C. glutamicum* possesses remarkable metabolic flexibility in how it allocates carbon and redox resources to support NADPH-intensive product formation, adding to our current understanding of pathway use in this microbe [29, 37–43]. Although AVD-3 and AVD-11 must meet the same precursor and NADPH demands, they operate using distinct central flux regimes. Both exhibited reduced biomass yields, leaving more carbon available for oxidation via pyruvate dehydrogenase and the early TCA cycle. This intensified TCA cycling increased NADPH generation via isocitrate dehydrogenase but also elevated CO_2_ release, consistent with overflow-like behavior reported for *C. glutamicum* on suboptimal carbon sources [28, 32, 33].

GapN fundamentally changed this balance. By providing NADPH without carbon loss, it reduced the dependence on the oxidative PP pathway and diminished the need for compensatory TCA cycling. As a result, AVD-11 generated NADPH at a markedly lower carbon cost than AVD-3. Quantifying the costs revealed that AVD-11 formed 1.27 NADPH per CO_2_ , almost 30% more than AVD-3 (1.0 NADPH per CO_2_) (Fig. 7C). Thus, the GapN-dependent redistribution of fluxes not only increased the amount of NADPH available for 5-AVD synthesis but also improved its carbon economy, reducing decarboxylation-derived losses. More broadly, these findings illustrate that high NADPH fluxes in *C. glutamicum* can be sustained without relying on a high PP pathway flux, an important principle for designing cell factories for other NADPH-intensive products [30, 31, 44]. Interestingly, the markedly reduced competition for carbon and NADPH from biomass formation—because both 5-AVD producers grew more poorly than LYS-12—did not translate into proportionally higher 5-AVD fluxes. Although more precursor carbon and reducing power were theoretically available for product synthesis, both 5-AVD strains were unable to exploit this relaxed anabolic demand to the extent possible. This mismatch indicates that additional bottlenecks remain limiting, underscoring that significant optimization potential still exists in these strains.

### Transport engineering highlights LysE as a flux gatekeeper

Efficient secretion is essential for maintaining 5-AVD flux and product selectivity, and our data identify LysE—the major lysine exporter of *C. glutamicum*—as a central control point. In LYS-1–based low producers, deletion of *lysE* abolished secretion of both lysine and 5-AVD, demonstrating that the pathway depends on LysE-mediated efflux for activity. In contrast, deletion in high-producing backgrounds reduced—but did not eliminate—5-AVD secretion, indicating the presence of secondary export routes. Overexpression of *lysE* shifted product selectivity toward lysine, consistent with intracellular lysine availability determining whether the precursor is exported directly or converted enzymatically to 5-AVD. Together, these results establish LysE as a flux “gatekeeper” that controls the balance between precursor secretion and derivative formation.

Attempts to expand export capacity using heterologous GABA permeases did not improve 5-AVD secretion. Given the structural analogy between 5-AVA and 5-AVD, testing *gabP* permeases was reasonable, as *gabP* expression had previously enhanced 5-AVA secretion in other hosts [18]. However, GABA permeases preferentially recognize substrates with a terminal carboxylate group and show low affinity for amide analogs [25, 45], which likely explains their lack of effect on 5-AVD export. In contrast, exporters of the LysE/RhtB family mediate efflux of basic amino acids and related amine metabolites [24, 46], making them more plausible candidates for 5-AVD transport. Transporters specific for 4-aminobutyramide or structurally related small primary amides could, in principle, also facilitate 5-AVD export, but to date no such bacterial transporters have been biochemically characterized so far.

**Fig. 9:**
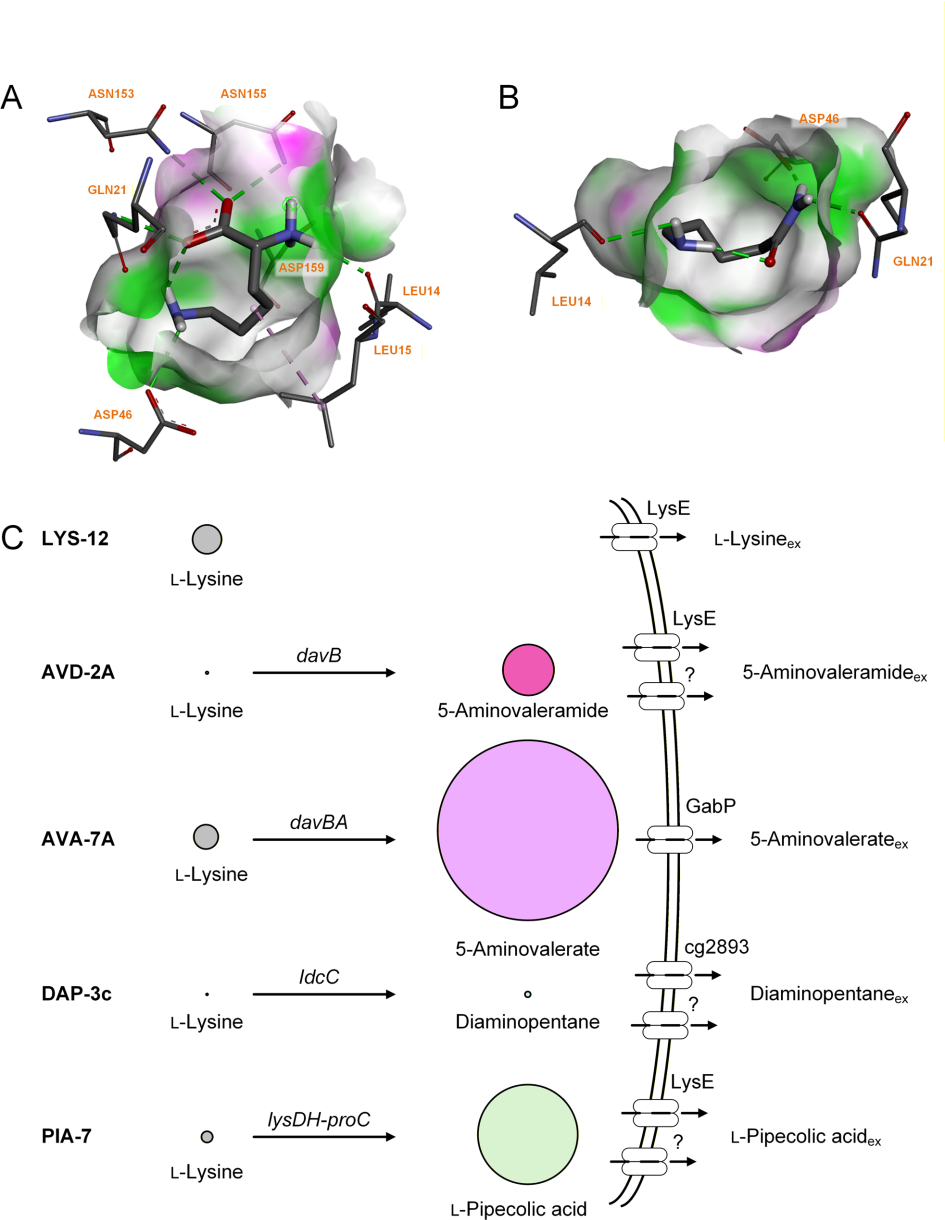
Intracellular metabolite pools and LysE substrate-binding poses in engineered *C. glutamicum* strains. Panels A and B show the predicted binding poses of lysine (**A**) and 5-AVD (**B**) within the LysE cavity, illustrating ligand placement, interactions with key binding-site residues, and the slight conformational adjustments that stabilize the bound configurations. Panel C presents metabolomics data for the lysine producer LYS-12 [14, 18] and several LYS-12–derived strains, namely AVD-2A producing 5-AVD (this work), AVA-7 producing 5-AVA [2], DAP-3c producing diaminopentane [20], and PIA-7 producing pipecolic acid [18]. The displayed values represent intracellular levels of lysine and the corresponding non-natural lysine derivatives, visualized as circles with areas proportional to metabolite concentration. The presently known exporters relevant for product secretion are indicated

To rationalize the observed export hierarchy across related products, we performed structure-guided substrate–transporter modelling using CB-Dock2 (Vina scoring) and CaverDock (tunnel-based transport-energy profiling) (Table 3). LysE displayed a clear predicted affinity hierarchy, binding fully protonated basic amino acids most strongly (ARG > PIP > LYS), whereas 5-AVD, 5-AVA and DAP also interact but more weakly (LYS > 5-AVD > AVA > DAP). CaverDock profiles confirmed the same trend. The resulting binding poses of lysine and 5-AVD illustrate ligand placement within the LysE cavity (Fig. 9A, B). These poses also reveal interactions with key binding-site residues previously identified as critical for LysE substrate recognition [47]. Consistent with their differing affinities, lysine engages seven of these key residues, whereas 5-AVD contacts only three, reflecting a substantially less extensive interaction network. Notably, both substrates undergo a slight conformational change upon binding so that their positively and negatively charged groups move closer together—which appears to stabilize the bound configuration through more favorable local electrostatic interactions. However, lysine consistently binds more strongly and occupies the pocket more complementarily than 5-AVD, indicating that lysine will outcompete 5-AVD for LysE access whenever both intracellular pools are high. This competitive advantage provides a mechanistic basis for the gatekeeper role of LysE: its preference for lysine over 5-AVD directly shapes the timing and selectivity of product release, explaining both the lysine-dominated export in *lysE*-overexpressing strains and the delayed onset of 5-AVD secretion during production. Furthermore, these modelling results align with the physiological observation that 5-AVD export is only partially reduced in *ΔlysE* strains, supporting the presence of a secondary, lower-affinity export route alongside LysE.

**Table 3:** Predicted binding affinities (AutoDock Vina scores) and CaverDock transport-energy minima for lyse with selected substrates

Substrate	Vina score (kcal/mol)	CaverDock minimum energy (kcal/mol)
Arginine (ARG)	− 6.3	− 5.1
Pipecolate (PIP)	− 5.6	− 4.7
Lysine (LYS)	− 5.4	− 4.1
5-Aminovaleramide (5-AVD)	− 4.6	− 3.7
5-Aminovalerate (AVA)	− 4.5	− 3.8
Diaminopentane (DAP)	− 3.8	− 3.2

More negative values indicate stronger predicted binding or lower energy along the transport tunnel. Data were obtained using CB-Dock2 and CaverDock based on the AlphaFold LysE structure

These findings align with broader industrial experience, where transport frequently limits microbial production [48]. Comparisons across LYS-12–derived strains reveal substantial differences in intracellular product accumulation (Fig. 9) Diaminopentane producers such as *C. glutamicum* DAP-3c [49] accumulate far less product than 5-AVD producers. In contrast, pipecolate and 5-AVA producers including PIA-7 [18] and AVA-7 [2] accumulate markedly higher intracellular concentrations, reflecting both strong pathway flux and limited recognition by available exporters. Intracellular pools also depend on pathway regulation: as little as 580 mg L^−1^ 5-AVD (5 mM) reduces DavB activity by > 40% in vitro, explaining its low steady-state levels and the limited benefit of stronger DavB expression [50]. Inefficient export can amplify such effects, leading to product accumulation, feedback inhibition and stress [1]. In *C. glutamicum*, secretion of non-natural molecules likely relies on multiple promiscuous transporter families, yet the responsible exporters remain largely unidentified [49].

Expanding and tailoring transporter repertoires will therefore be essential for engineering robust cell factories for non-natural lysine derivatives. For 5-AVD, promising strategies include fine-tuned *lysE* expression, rational LysE engineering [47], or identification of native exporters with higher affinity [51]. Finally, the host’s inability to reimport 5-AVD once secreted represents a practical advantage, preventing futile cycling and enabling stable extracellular accumulation [1].

### Process performance and industrial relevance of 5-AVD production

In fed-batch cultivation on sucrose–molasses medium, the baseline genomic producer AVD-2A and the optimized strain AVD-11 were benchmarked to assess process robustness and scalability. Both strains displayed strong growth and robust utilization of the complex feedstock. AVD-11 outperformed the baseline, achieving titers above 36 g L^−1^ and a maximum space–time yield of 1.2 g L^−1^ h^−1^. Importantly, product selectivity exceeded 97% for both strains, with only trace amounts of lysine detected. These values are on par with, or surpass, established microbial routes to other lysine derivatives, underlining the promise of 5-AVD as a scalable bio-based chemical. The robustness of production on molasses, the low by-product profile, and the autonomous DO-based feeding strategy underscore the potential to scale this process towards industrial feasibility. Molasses appears as a relevant substrate, given its worldwide availability [52, 53] and process suitability, with *C. glutamicum* efficiently converting high sugar levels (80 g L^−1^). Moreover, the absence of reuptake systems and the high tolerance of *C. glutamicum* to 5-AVD (50 g L^−1^) facilitate accumulation at high titers.

A key requirement for industrial implementation is the genetic stability of production hosts under extended high-density cultivation. The chromosomal integration strategy applied in this study proved advantageous in this regard. PCR analysis confirmed that the *davB* cassette remained intact throughout the entire fed-batch process in both AVD-2A and AVD-11, demonstrating that the engineered pathway is stably maintained even under strong selective pressure toward high flux. This stability is consistent with the uniform production profiles observed in fed-batch fermentation and indicates that neither construct loss nor rearrangements contribute to the modest decline in productivity seen late in the process, supporting the suitability of the created cell factories for scale-up and further process intensification.

Future optimization to push productivity and titers toward industrial benchmarks might include optimizing nitrogen feeding to sustain high fluxes, developing continuous or high-cell-density fermentation strategies, and tailoring downstream processing for cost-effective recovery. For downstream processing, the high selectivity of 5-AVD secretion is advantageous, likely reducing purification costs. Approaches such as ion-exchange chromatography [54] or crystallization can be adapted for recovery at scale [55]. Beyond process intensification, techno-economic assessment will be valuable to quantify the competitiveness of microbial 5-AVD against petrochemical routes [56].

Establishing a scalable microbial route to 5-AVD expands the portfolio of bio-based building blocks accessible from lysine. As discussed above, 5-AVD serves as a versatile precursor not only for polyamides such as nylon-5 but also for a wide range of amide-containing fine chemicals and bioactive molecules. Its direct production from renewable sugars thus provides a sustainable alternative to petrochemical synthesis while simultaneously enabling new applications in polymer materials [57], drug delivery systems [58], 3D-printable tissue engineering devices [59], biodegradable surfactants [60], and pharmaceutical scaffolds [61] based on amide chemistry. Furthermore, 5-AVD might be interesting as a noncanonical amino acid building block in antimicrobial peptides [62].

## Conclusions

This study establishes *C. glutamicum* as an efficient microbial platform for the biosynthesis of 5-aminovaleramide (5-AVD), a promising bio-based monomer and versatile chemical intermediate. Through successive rounds of metabolic engineering, we show that 5-AVD production can be strongly enhanced by optimizing precursor supply, balancing redox cofactors, and fine-tuning export capacity. In particular, redox engineering via GapN enabled yields above 0.30 mol mol^−1^ glucose with complete selectivity for 5-AVD, while fed-batch benchmarking confirmed the industrial robustness of the process, achieving titers above 36 g L^−1^ with excellent product selectivity (> 97%). Our findings highlight the critical role of LysE as a flux gatekeeper in balancing lysine export and 5-AVD conversion and underscore the limitations of current transporter engineering approaches.

Overall, this work provides both proof of concept and a foundation for further development of sustainable 5-AVD production. Future improvements in enzyme activity, transporter specificity, redox metabolism, and process intensification will be key to advancing 5-AVD toward industrial application. More broadly, the strategies applied here contribute to expanding the repertoire of lysine derivatives accessible via microbial cell factories, strengthening the role of *C. glutamicum* as a versatile chassis for bio-based monomer production.

## Materials and methods

### Microorganisms, plasmids, and genes

The bacterial strains *C. glutamicum* LYS-12, *C glutamicum* LYS-1 [14], *C. glutamicum* LYS-1 Δl*ysE* [19], and *C. glutamicum* LYS-12 *P*_*tuf*_ *gapN* [33] were obtained from previous studies. *Escherichia coli* DH10B and NM522 served as host strains for plasmid amplification and methylation [14]. The native lysine 2-monooxygenase (*davB*) gene was isolated from *Pseudomonas putida* KT2440. The codon-optimized variant of *davB* (synthesized by Eurofins Genomics, Ebersberg, Germany) and GABA permease genes from *E. coli* K12 and *Mycobacterium smegmatis* (synthesized by GenScript, Piscataway Township, NJ, USA) were designed based on digital sequence data. The mutagenized *sod*^*opt2*^ promoter was obtained from previous research [18]. Genetic modifications were introduced using either the integrative suicide plasmid *pClik int sacB* [28] or the self-replicating plasmid *pClik5a MCS* [40]. To ensure proper methylation patterns for corynebacterial transformation, co-expression of the *pTC* plasmid in *E. coli* NM522 was performed [14]. Table 4 summarizes all strains and plasmids used in this study, and primers are listed in Additional File 1, Table S4.

**Table 4:** Strains and plasmids used in this study

Strain/Plasmid	Description	References
*E. coli*		
DH10B	Cloning host	Invitrogen
NM522	Cloning host	Invitrogen
*C. glutamicum*		
LYS-12	l-lysine hyperproducer with 12 genomic modifications based on *C. glutamicum* ATCC13032.	[14]
LYS-1	*C.glutamicum* ATCC13032 with the genomic modification C932T in *lysC*	[14]
LYS-1 Δ*lysE*	*C. glutamicum* LYS-1 with partial deletion of the lysine exporter *lysE*	[19]
LYS-12 *P*_*tuf*_ *gapN*	*C. glutamicum* LYS-12 with genome-based expression of codon-optimized *gapN* from *S. mutans* UA159 under control of *P*_*tuf*_	[31]
LYS-12 *P*_*tuf*_ *davBA*^*opt*^	LYS-12 with genomic expression of the monocistronic module *P*_*tuf*_ *davBA* using codon-optimized *P. putida* genes	[2]
AVD-1A	*C. glutamicum* LYS-12 with self-replicating *pClik5α P*_*tuf*_ *davB*^*nat*^	This work
AVD-1B	*C. glutamicum* LYS-12 with self-replicating *pClik5α P*_*tuf*_ *davB*^*opt*^	This work
AVD-2A	*C. glutamicum* LYS-12 with genome-based expression of native *davB* from *P. putida* KT2440 under control of *P*_*tuf*_.	This work
AVD-2B	*C. glutamicum* LYS-12 with genome-based expression of codon-optimized *davB* from *P. putida* KT2440 under control of *P*_*tuf*_.	This work
AVD-3	*C. glutamicum* LYS-12 with genome-based expression of *davB* from *P. putida* KT2440 under control of *P*_*sod*_^*opt2*^.	This work
AVD-4A	*C. glutamicum* LYS-1 with self-replicating *pClik5α P*_*tuf*_ *davB*^*nat*^	This work
AVD-4B	*C. glutamicum* LYS-1 Δ*lysE* with self-replicating *pClik5α P*_*tuf*_ *davB*^*nat*^	This work
AVD-5A	*C. glutamicum* LYS-1 with self-replicating *pClik5α P*_*tuf*_ *davB*^*opt*^	This work
AVD-5B	*C. glutamicum* LYS-1 Δ*lysE* with self-replicating *pClik5α P*_*tuf*_ *davB*^*opt*^	This work
AVD-6A	*C. glutamicum* AVD-2A with partial deletion of the lysine exporter *lysE*	This work
AVD-6B	*C. glutamicum* AVD-2B with partial deletion of the lysine exporter *lysE*	This work
AVD-7A	*C. glutamicum* AVD-2A with genome-based expression of GABA permease gabPIII (PP2911) from *P. putida* KT2440 undercontrol of *P*_*tuf*_	This work
AVD-7B	*C. glutamicum* AVD-3 with genome-based expression of GABA permease gabPIII (PP2911) from *P. putida* KT2440 under control of *P*_*tuf*_	This work
AVD-8A	*C. glutamicum* AVD-2A with genome-based expression of GABA permease *gabP* I from *M. smegmatis* under control of *P*_*tu*f_	This work
AVD-8B	*C. glutamicum* AVD-2A with genome-based expression of the second GABA permease *gabP* II from *M. smegmatis* under control of *P*_*tuf*_	This work
AVD-9A	*C. glutamicum* AVD-2B with genome-based expression of GABA permease *gabP* I from *M. smegmatis* under control of *P*_*tuf*_	This work
AVD-9B	*C. glutamicum* AVD-2B with genome-based expression of GABA permease *gabP* from *E. coli* under control of *P*_*tuf*_	This work
AVD-10	*C. glutamicum* LYS-12 with genome-based expression of *davB* from *P. putida* KT2440 under control of *P*_*tuf*_ and genome-based expression codon-optimized *gapN* from *S. mutans* UA159 under control of *P*_*tuf*_	This work
AVD-11	*C. glutamicum* LYS-12 with genome-based expression of *davB* from *P. putida* KT2440 under control of *P*_*sod*_^*opt2*^ *and* genome-based expression codon-optimized *gapN* from *S. mutans* UA159 under control of *P*_*tuf*_.	This work
AVD-12	*C. glutamicum* LYS-12 with genome-based expression of *davB* from *P. putida* KT2440 under control of *P*_*sod*_^*opt2*^ and overexpression of *lysE* using *P*_*sod*_.	This work
Plasmids		
*pTC*	Episomal vector for the expression of the methyltransferase gene of *C. glutamicum*, *ORI* of *E. coli*, *MCS*, *tetR*	[14]
*pClik5a MCS*	Episomal vector for the expression in *C. glutamicum*, *ORI* for *E. coli* and *C. glutamicum*, *MCS*, *kanR*	[14]
*pClik5α P*_*tuf*_ *davB*^*nat*^	Episomal vector for the expression of the native *davB* gene from *P. putida* KT2440 under control of the promoter *P*_*tuf*_	This work
*pClik5α P*_*tuf*_ *davB*^*opt*^	Episomal vector for the expression of the codon-optimized *davB* gene from *P. putida* KT2440 under control of the promoter *P*_*tuf*_	This work
*pClik int sacB*	Transformation vector for genetic modifications by homologous recombination in *C. glutamicum*; *ORI* of *E. coli*; *MCS*, *kanR*, *sacB*	This work
*pClik int sacB P*_*tuf*_ *davB*^*Pput−nat*^ (*bioD*)	Transformation vector for the integration of the native *davB* gene from *P. putida* KT2440 under control of *P*_*tuf*_ into the *bioD* gene locus	This work
*pClik int sacB P*_*tuf*_ *davB*^*Pput−opt*^ (*bioD*)	Transformation vector for the integration of a codon-optimized variant of *davB* from *P. putida* KT2440 under control of *P*_*tuf*_ into the *bioD *gene locus	This work
*pClik int sacB P*_*sod*_^*opt2*^ *davB*^*Pput−nat*^ (*bioD*)	Transformation vector for the integration of the native *davB* gene from *P. putida* KT2440 under control of *P*_*sod*_^*opt2*^ into the *bioD* gene locus	This work
*pClik int sacB* Δ*lysE*	Transformation vector for the partial deletion of *lysE* gene	[49]
*pClik int sacB P*_*tuf*_ *PP2911* (*gapTDP*)	Genomic replacement of *gabTDP* by *P*_*tuf*_ *gabPIII* PP2911	[2]
*pClik int sacB P*_*tuf*_ *gabP*^*Ecol*^ (*crtB*)	Transformation vector for the integration of a copy of the *gabP* gene from *E. coli* K12 under control of *P*_*tuf*_ into the *crtB* gene locus	This work
*pClik int sacB P*_*tuf*_ *gabP*^*Msme1*^ (*crtB*)	Transformation vector for the integration of a copy of the *gabP* I gene from *M. smegmatis* under control of *P*_*tuf*_ into the *crtB* gene locus	This work
*pClik int sacB P*_tuf_*gabP* ^*Msme2*^ (*crtB*)	Transformation vector for the integration of a copy of the *gabP* II gene from *M. smegmatis* under control of *P*_*tuf*_ into the *crtB* gene locus	This work
*pClik int P*_*sod*_ *lysE*	Overexpression of the lysine exporter gene *lysE* by integration of the promoter *P*_*sod*_ upstream *lysE*	This work

### Molecular and genetic engineering

Cloning strategies were designed in silico using SnapGene software (Version 5.3.2, GSL Biotech, Chicago, IL, USA). Plasmid DNA synthesis, purification, and analysis were carried out as described previously [1]. Specific DNA fragments were amplified from genomic DNA by PCR using designated primers and the Phusion High-Fidelity PCR Master Mix with HF Buffer (New England Biolabs, Frankfurt am Main, Germany). Linearized vector backbones (*Sma*I, *Eco*RV) were assembled with amplified DNA fragments through Gibson assembly. The resulting plasmids were introduced into *E. coli* via heat shock transformation and into *C. glutamicum* via electroporation [63]. Correct transformants were verified using colony PCR (Phire Green Hot Start II PCR Master Mix, Thermo Fisher Scientific) and confirmed by Sanger sequencing (Azenta, Chelmsford, MA, USA).

### Genetic constructs for 5-aminovaleramide synthesis

The synthesis of 5-aminovaleramide (5-AVD) was achieved by introducing the *davB* gene from *P. putida* KT2440 under the control of the constitutive *tuf* promoter [28] or the mutagenized *sod*^*opt2*^ promoter [18]. Expression was carried out using either the self-replicating plasmid *pClik5a MCS* or a genome-integrated construct based on *pClik int sacB*. Additionally, for genome-based expression of heterologous GABA permeases under the *tuf* promoter, as well as targeted deletion and overexpression of *lysE*, the *pClik int sacB* vector was utilized for DNA transfer into *C. glutamicum*.

To confirm the genetic stability of the genome-integrated *davB* constructs during fed-batch fermentation, PCR analyses were performed on strains AVD-2A and AVD-11 before inoculation and after completion of the fermentation process (see below). Primers were designed to anneal 998 bp upstream and 954 bp downstream of the *bioD* integration locus, yielding expected amplicon sizes of 3,835 bp for AVD-2A and 3,827 bp for AVD-11.

### Growth conditions

Cultures of *E. coli* and the first pre-culture of *C. glutamicum* were grown in BHI medium (37 g L⁻^1^ Brain Heart Infusion, Becton Dickinson, Heidelberg, Germany). The second pre-culture and main culture of *C. glutamicum* were conducted in a chemically defined minimal medium containing per liter: 10 g glucose, 15 g (NH_4_)_2_SO_4_, 100 mL 2 M potassium phosphate buffer (pH 7.8), 1 g NaCl, 0.2 g MgSO_4_·7H_2_O, 55 mg CaCl_2_, 20 mg FeSO_4_·7H_2_O, 0.5 mg biotin, 1 mg thiamin·HCl, 1 mg calcium pantothenate, 30 µg 3,4-dihydroxybenzoic acid, and 10 mL of a 100x trace element solution (200 mg L⁻^1^ FeCl_3_·6H_2_O, 200 mg L⁻^1^ MnSO_4_·H_2_O, 50 mg L⁻^1^ ZnSO_4_·7H_2_O, 20 mg L⁻^1^ CuCl_2_·2H_2_O, 20 mg L⁻^1^ Na_2_B_4_O_7_·10H_2_O, 10 mg L⁻^1^ (NH_4_)_6_MO_7_O_24_·4H_2_O, pH 1.0). For plasmid maintenance, the medium was supplemented with 50 µg mL^−1^ kanamycin or 12.5 µL mL^−1^ tetracycline. The liquid medium was solidified with 20 g L^−1^ Difco agar (Becton Dickinson).

Single colonies from BHI agar plates (pre-incubated for 48 h at 30 °C) were used to inoculate the first pre-culture in baffled shake flasks (10% filling volume), which was grown overnight on a rotary shaker (30 °C, 230 rpm, 85% humidity, Multitron, Infors AG, Bottmingen, Switzerland). Cells were then harvested (3 min, 8800×*g*, room temperature) and used to inoculate the second pre-culture (baffled shake flasks, 10% filling volume). The main culture was conducted in biological triplicates and incubated under identical shaking conditions. Mid-exponentially grown cells from the second pre-culture were transferred into fresh main cultivation medium (50 mL medium in 500 mL baffled shake flasks).

Screening of *C. glutamicum* strains was performed using microtiter plates (48-well flower plates, Beckman Coulter GmbH, Baesweiler, Germany) in a mini-bioreactor system as previously described (1300 rpm, 30 °C, 85% humidity, Beckman Coulter GmbH) [64]. All experiments were conducted in biological triplicate to ensure statistical significance.

### Fed-batch cultivation in stirred tank bioreactors

The production performance of 5-AVD-producing strains was evaluated in a fed-batch process. Strains were grown in 1 L lab-scale bioreactors, monitored and controlled using the DASGIP control software (SR0700ODLS, Eppendorf, Hamburg, Germany). The initial batch medium (300 mL) contained: 70 g L^−1^ sugar beet molasses (Pfeifer & Langen, Köln, Germany), 50 g L^−1^ sucrose, 15 g L^−1^ yeast extract (Becton Dickinson), 10 mL L^−1^ corn steep liquor (Selfmade-Baits, Brandenburg, Germany), 35 g L^−1^ (NH_4_)_2_SO_4_, 2 g L^−1^ citric acid, 1.25 g L^−1^ KH_2_PO_4_, 1.25 g L^−1^ Na_2_HPO_4_, 1.25 g L^−1^ MgSO_4_·7H_2_O, 35 mg L^−1^ FeSO_4_·7 H_2_O, 35 mg L^−1^ FeCl_3_·6H_2_O, 30 mg L^−1^ of ZnSO_4_·7H_2_O, 168 mg L^−1^ of CaSO_4_·2H_2_O, 9.1 mg L^−1^ of MnSO_4_·H_2_O, 15 mL of a vitamin solution (300 mg L^−1^ biotin, 500 mg L^−1^ thiamin·HCl, 600 mg L^−1^ nicotinamide, 2 g L^−1^ calcium pantothenate), 1.43 mL of a trace element solution (2.1 g L^−1^ citric acid, 300 mg L^−1^ boric acid 456 mg L^−1^ CuSO_4_·5H_2_O, 428 mg L^−1^ CoSO_4_·7H_2_O, 338 mg L^−1^ NiSO_4_·6H_2_O, and 59 mg L^−1^ Na_2_MoO_4_·2H_2_O) and 2 mL L^−1^ antifoam.

Prior to the process, cells for the initial preculture were grown overnight at 30 °C in 25 mL of BHI medium supplemented with 40 g L^−1^ glucose· H_2_O and 10 g L^−1^ yeast extract. Then second precultures were prepared by transferring first precultures to 1 L baffled shake flasks and adding 75 mL of the same medium (pre-warmed to 30 °C). After incubation for 8 h cells were harvested (2 min, 5000×*g*, room temperature) and used as inoculum for 300 mL of the batch medium. During fermentation, temperature was maintained at 30 °C (CWD4 Bioblock, Eppendorf), while the pH was kept at 7.0 ± 0.1 using a pH probe (405-DPAS–SC–K8S/225, Mettler Toledo, Giessen, Germany) and the automatic addition of 6 M NaOH or HCl (MP8 pump system, Eppendorf). The dissolved oxygen level was monitored by online measurement by a pO_2_ electrode (VisiFerm DO 225, Hamilton, Höchst, Germany) and maintained above a saturation of 40% by increasing stirrer speed (400 rpm up to 1600 rpm), O_2_ content (21 to 100%), and aeration rate (18 to 50 sL h^−1^). Additionally, the composition of exhaust gas (O_2_, CO_2_) was analyzed throughout the process (GA4, Eppendorf). After the batch phase, pulses of 10 mL feed solution (115 g L^−1^ sugar beet molasses (Pfeifer & Langen, Köln, Germany), 515 g L^−1^ sucrose, 210 g L^−1^ (NH_4_)_2_SO_4_, 22.5 mL L^−1^ vitamin solution, 2.145 mL L^−1^ trace element solution and 3 mL L^−1^ antifoam) were added when a sudden increase in pO_2_ to more than 40% occurred.

### Determination of cell density

Cell density was quantified by measuring the optical density at 660 nm (OD_660_) using a spectrophotometer. A previously established correlation factor was used to calculate the corresponding cell dry weight (CDW) [1].

### Quantification of substrates and intermediates

Glucose concentrations were determined via high-performance liquid chromatography (HPLC) using the 1260 Infinity Series system (Agilent Technologies). An isocratic separation was performed on an Aminex HPX-87 H column (7.8 mm × 300 mm × 9 μm, Bio-Rad Laboratories, Hercules, CA, USA) at 55 °C. The mobile phase consisted of 3.5 mM H_2_SO_4_ with a flow rate of 0.8 mL min^−1^ [1]. Detection was conducted using a refractive index detector (1260 RID, G1362A, Agilent Technologies) calibrated with external standards.

The concentration of 5-aminovaleramide was measured by HPLC (1260 Infinity Series, Agilent) using a C18 column (4.6 mm × 100 mm × 3.5 μm, Gemini Phenomenex, Torrance, CA, USA) at 40 °C. The mobile phase consisted of a gradient of eluent A (20 mM NaH_2_PO_4_, 0.5 g L^−1^ sodium azide, pH 7.8) and eluent B (45% acetonitrile, 45% methanol, 10% water) with a flow rate of 1 mL min^−1^. The gradient program was as follows: 0 min: 75% A, 0–14 min: 61% A, 14.0–14.5 min: 0% A, 14.5–16.5 min: 0% A, 16.5–17 min: 75% A, 17–19 min: 75% A. Fluorescence detection was performed at excitation/emission wavelengths of 340/450 nm using a fluorescence detector (G1321A, Agilent Technologies). Lysine quantification was carried out as described previously [65]. α-Aminobutyric acid (220 µM) was used as an internal standard.

### Structural identification of 5-aminovaleramide using GC-MS

To confirm the identity of 5-aminovaleramide (5-AVD), gas chromatography-mass spectrometry (GC-MS) analysis was performed. Cultivation supernatant from *C. glutamicum* LYS-12 *P*_*tuf*_ *davBA*^*opt*^ (20 µL) was dried under a stream of nitrogen (N₂) for approximately 20 min. The dried sample was then derivatized by adding a mixture of 50 µL dimethylformamide containing 0.1% pyridine and 50 µL *N*-methyl-*N*-*tert*-butyldimethylsilyl-trifluoroacetamide (MBDSTFA) (Macherey-Nagel, Düren, Germany), followed by incubation at 80 °C for 30 min [32]. Prior to GC-MS analysis, salts and other interfering particulates were removed by centrifugation (10 s, 2000×*g*, Sprout Mini Centrifuge, Heathrow Scientific, Vernon Hills, IL, USA).

The GC-MS setup comprised a gas chromatograph (GC 7890 B, Agilent Technologies) equipped with an HP-5MS column (5% phenyl-methylpolysiloxane, 30 m × 250 μm × 0.25 μm, Agilent Technologies) and helium (He 5.0) as the carrier gas (1.2 mL min^−1^). The system included a quadrupole mass spectrometer detector (MSD 5977 A, Agilent Technologies) for compound identification. A 1 µL sample was injected with a split ratio of 1:30. The column temperature program was as follows: an initial temperature of 50 °C held for 2 min, followed by a ramp of 8 °C min^−1^ to 200 °C (2–12 min), and then an increase of 10 °C min^−1^ to a final temperature of 325 °C (12–27 min). Additional instrument settings included an inlet temperature of 325 °C, an ion source temperature of 230 °C, an interface temperature of 325 °C, and a quadrupole temperature of 150 °C. Due to insufficient identification of the analyte through library searches using the MassHunter Library (NIST08.L, Agilent Technologies), an additional isotopic labeling experiment was conducted. Fully labeled [^15^N] ammonium sulfate (Sigma-Aldrich) was used in place of naturally labeled nitrogen sources during cultivation. Cells were harvested at mid-exponential and late-exponential growth phases (3 min, 4 °C, 17,000×*g*). The relative mass isotopomer distribution of the ion cluster [M-57]^+^ containing all analyte carbon [66] was corrected using established methods [67] for further structural analysis and validation of 5-AVD identity.

### Analysis of the enzymatic activity of lysine 2-monooxygenase

Exponentially growing cells (50 mL) in biological triplicates were harvested by centrifugation (8200×*g*, 4 °C, 5 min), washed, and resuspended in 4 mL cold phosphate buffer (100 mM, pH 7.8). Aliquots of 1 mL were transferred to lysing matrix B tubes (MP Biomedicals, Eschwege, Germany) and disrupted using a Ribolyser (Precellys 24, Bertin Technologies, Ile de France, France) in two cycles at 5500×*g* for 30 s, with a 2-min cooling step between cycles. Cell debris was removed by centrifugation (10,000×*g*, 4 °C, 20 min). Enzymatic activity was measured in technical triplicates using 10 mL open baffled shake flasks incubated in an orbital shaker (30 °C, 85% humidity, 230 rpm, rotation radius 5 cm, HT Infors Multitron, Bottmingen, Switzerland). The reaction was initiated by adding 500 µL of cell extract to 9.5 mL of a prepared reaction mixture containing 20 mM lysine and 100 mM phosphate buffer (pH 7.8). At predetermined time points, the reaction was terminated by heating at 100 °C for 5 min. Samples were subsequently centrifuged and stored at − 20 °C until quantification via HPLC. Lysine consumption was equated to the enzymatic activity of lysine 2-monooxygenase [2]. Negative controls without enzyme extract or substrate were included to validate the assay. The total protein concentration in the cell extract was estimated using the Bradford method (Protein Assay Dye Reagent Concentrate, Bio-Rad Laboratories) with bovine serum albumin as the standard [68].

### Quantification of the enzymatic activity of NADP^+^-dependent glyceraldehyde 3-phosphate dehydrogenase

Cell extracts were prepared as described previously [33]. The activity of NADP^+^-dependent glyceraldehyde 3-phosphate dehydrogenase (GapN) was quantified by monitoring changes in NADPH absorption at 340 nm [31].

### Extraction and quantification of intracellular metabolites

Intracellular metabolites (amino acids, 5-AVD, diaminopimelate) were extracted using rapid filtration followed by sample boiling [69]. Quantification was performed via HPLC [70]. A correlation factor of 1.95 (µL cytoplasm) mg_CDW_^−1^ was applied for final calculations [38].

### Extraction and quantification of NADP^+^ and NADPH

The NADP⁺/NADPH ratio in selected strains was determined using the NADP⁺/NADPH Assay Kit (ab176724, Abcam, Cambridge, UK). Reagents and materials were prepared following the manufacturer’s protocol [18]. Cell cultures were cultivated under standard conditions, harvested at a density of 1.5 × 10^7^ cells by centrifugation (10,200×*g*, 4 °C, 15 min), and subsequently lysed. Following the manufacturer’s instructions, NADPH, NADP⁺, and total redox equivalents were extracted and analyzed using a fluorometric assay (540/590 nm, BioTek Synergy H1 Multimode Reader, Agilent Technologies, Santa Clara, CA, USA). The total NADP⁺/NADPH and NADPH concentrations were calculated using a calibration curve generated from serial dilutions of NADPH stock solutions in phosphate-buffered saline (PBS; 137 mM NaCl, 2.7 mM Na_2_HPO_4_, 18 mM KH_2_PO_4_, pH 7.4).

### Quantitative PCR (qPCR)

RNA was extracted and purified using the RNeasy Mini Kit (Qiagen, Hilden, Germany) with on-column DNase digestion (RNase-Free DNase Set, Qiagen). Then, total RNA concentration was quantified (NanoDrop 1000 spectrophotometer, peqlab, Erlangen, Germany), and RNA quality was evaluated with the RNA 6000 Nano Kit (2100 Bioanalyzer System, Agilent Technologies). All samples had an RNA integrity number (RIN) higher than 9.3. RNA samples were converted to first-strand cDNA using the Maxima First Strand cDNA Synthesis Kit with dsDNase (Thermo Fisher, Waltham, MA, USA). Primer pairs for qPCR targeting the *gabP* gene variants and *davB* were designed using Primer-BLAST [71], whereas primers for the housekeeping gene *sigA* were adopted from previously validated assays [72] (Additional File 1, Table S4). qPCR was carried out on a QuantStudio™ 3 Real-Time PCR System (Applied Biosystems, Thermo Fisher Scientific) using PowerUp SYBR Green Master Mix (Thermo Fisher Scientific). Data processing, including normalization to *sigA*, was performed using the QuantStudio Design and Analysis Software (Applied Biosystems, Thermo Fisher Scientific).

### Isotopic tracer studies and ^13^C labeling analysis

Two precultures were performed as for the other cultivations. First, *C. glutamicum* cells were grown in complex medium (BHI) and then transferred to a second preculture in minimal medium already containing the respective ¹³C tracer glucose to be used in the main experiment [73]. Cells from the second preculture were harvested and transferred to 50 mL of ^13^C tracer medium. The initial cell concentration was kept below an OD_660_ of 0.2 to avoid interference from unlabeled biomass during subsequent ^13^C labeling analysis [66]. To explore the metabolic network of *C. glutamicum*, we used parallel setups containing either [1-^13^C] glucose or a 1:1 mixture of naturally labeled glucose and [U-^13^C_6_] glucose as tracer substrates [74]. For determination of physiological parameters (growth, glucose consumption, and organic acid secretion), three additional biological replicates were performed on non-labeled glucose (Table 2). During exponential growth (OD_660_ 2–6), cells were harvested and processed for GC-MS-based ^13^C labeling analysis. This analysis focused on amino acids from cell protein [75] and secreted metabolites [76].

### Metabolic flux estimation

Metabolic fluxes were estimated based on the *C. glutamicum* network (Additional File 1, Table S5), stoichiometric growth data (Table 2) and data on anabolic precursor demand (Additional File 1, Table S6), using OpenFLUX software [77] integrated with MATLAB (MathWorks, Natick, USA) [78]. Before flux estimation, mass isotopomer distributions were corrected for naturally occurring isotopes [67] using a built-in algorithm. Due to the nonlinear nature of isotopomer models, 100 independent parameter estimations with random initial starting points were performed. All iterations converged to the same solution, confirming the robustness of the data and ensuring that the flux distribution corresponded to the global minimum [79]. After estimating flux parameters, 95% confidence intervals were determined through Monte Carlo analysis over 100 iterations [80]. Overall, the data showed an excellent fit to the metabolic model (Additional File1, Table S7). The ^13^C-flux analysis provided precise estimates for the total glyceraldehyde-3-phosphate dehydrogenase flux. In AVD-11, this total represented the combined activity of the native NAD⁺-dependent GAPDH and the NADP⁺-dependent GapN, but their individual contributions could be directly separated by ^13^C labeling alone. To resolve this, we used the balance of NADPH-supplying fluxes. In AVD-3, the summed NADPH-generating fluxes (oxidative PP pathway, malic enzyme, and isocitrate dehydrogenase) reached 250% relative to glucose uptake. Because AVD-3 and AVD-11 exhibited nearly identical growth rates and very similar 5-AVD production profiles, we assumed the same overall NADPH demand (250%) for AVD-11. For AVD-11, we first summed the measurable NADPH-forming fluxes from the oxidative PP pathway, malic enzyme, and the TCA cycle. The remaining difference to the total of 250% was then assigned to GapN. This procedure allowed quantitative estimation of the GapN-specific flux within the combined GAPDH/GapN node and enabled reconstruction of the full intracellular NADPH regeneration profile for both strains.

### Structural modelling and substrate-transporter docking

Structural modelling was performed to rationalize substrate interactions with the *C. glutamicum* LysE exporter. A four-step workflow was applied using the following tools: CB-Dock2 for cavity detection and docking [81], CaverDock for tunnel identification and transport-energy profiling [82], and BIOVIA Discovery Studio (Discovery Studio Modeling Environment, Release 2025 SP1; Dassault Systèmes BIOVIA, San Diego, CA, USA) for visualization. For protein structure analysis and cavity detection, a high-confidence structural model of LysE was obtained from the AlphaFold Protein Structure Database (entry AF-P94633-F1) and used as the receptor for all analyses. Putative ligand-binding cavities were identified using CB-Dock2, which automatically detects concave regions suitable for docking and prepares the corresponding search boxes. Among the detected cavities, we prioritized binding sites consistent with residues previously implicated in LysE substrate recognition [47]. Using these cavities, CB-Dock2 executed Autodocking Vina [83] in automated blind-docking mode against the following ligands: lysine, arginine, 5-AVD, pipecolate, and DAP. Vina scores (kcal mol^−1^) served as predicted binding affinities and reflect an empirical evaluation of steric complementarity, hydrophobic contacts, hydrogen bonding potential, and conformational entropy. It should be noted that the docking scores indicate potential interaction but do not confirm transport capability, transport directionality, or export rate. To assess substrate compatibility with the export pathway, transport tunnels within the LysE structure were identified using the CAVER algorithm integrated in CaverDock. For selected ligands, CaverDock simulated ligand movement along the tunnel axis and computed binding-energy profiles, including minimum-energy regions and putative energy barriers relevant to transport. The resulting docked poses, tunnel trajectories, and interaction geometries were inspected and visualized using Discovery Studio Visualizer to support qualitative interpretation and figure generation.

## Supplementary Information

Below is the link to the electronic supplementary material.

Supplementary Material 1. Table S1: Kinetics and stoichiometry of growth and product formation in C. glutamicum strains producing 5-aminovaleramide (5-AVD) with and without deletion of the l-lysine exporter lyse. Strains were based on the expression of native or codon-optimized *davB* from *P. putida* KT2440 under the constitutive promoter *P*_*tuf*_ in chassis strains LYS-1 and LYS-12. Cultivations were performed in mini-bioreactors on glucose minimal medium at 30 °C. Data include specific growth rates (µ) and yields (Y). Data represent mean ± standard error of three biological replicates. Table S2: Intracellular amino acid concentrations in 5-AVD-producing strains AVD-2A and AVD-2B, and their *lysE*-deficient derivatives AVD-6 A and AVD-6B. Strains were cultivated in baffled shake flasks on glucose minimal medium at 30 °C, and samples were taken during mid-exponential phase. Data represent mean ± standard error of three biological replicates. Table S3. Kinetics and stoichiometry of growth and product formation in *C. glutamicum* AVD-12, overexpressing *lysE* under the constitutive *sod* promoter in strain AVD-3. Cultivation was performed in glucose minimal medium at 30 °C in shake flasks. Data include the specific growth rate (µ), substrate consumption and product formation (q), and yields (Y). Data represent mean ± standard error of three biological replicates. Table S4: Primers used in this study. Table S5: Biochemical reaction network used for *C. glutamicum* AVD-3 and AVD-11. Table S6: Anabolic drain fluxes used for parameter estimation allowing a 10% deviation in biomass composition [84] and yield (Table 3). All data are given as relative flux (%) related to the specific glucose uptake rate. Table S7: Experimentally determined and simulated relative fractions of mass isotopomers. Figure S1: Isotopic labeling analysis of secreted metabolites in strain LYS-12 *P*_*tuf*_ *davBA*^*opt*^ cultivated in glucose minimal medium with fully ¹⁵N-labeled ammonium sulfate. Relative isotope abundances of major fragment ions ([M–57]⁺) were compared with commercial standards of 5-AVD, 5-AVA, and GTA. Mass shifts confirmed incorporation of nitrogen atoms (+ 2 Da for 5-AVD, + 1 Da for 5-AVA, + 0 Da for GTA). Figure S2: Inability of *C. glutamicum* LYS-12 to grow on 5-AVD as sole carbon source. Growth was monitored in mini-bioreactors with minimal medium containing 10 g L^− 1^ glucose, 4.6 g L^− 1^ 5-AVD, or both. No biomass formation occurred with 5-AVD alone. Data represent mean ± standard error of three biological replicates. Figure S3: Effect of 5-AVD on the growth of *C. glutamicum* LYS-12. Cells (initial OD_**660**_ = 1) were cultivated in a miniaturized bioreactor system on BHI medium supplemented with defined 5-AVD concentrations. Data represent mean ± standard error from three biological replicates (A, B). For growth on solid BHI agar, 5 µL of cell dilutions were spotted and incubated at 30 °C for 40 h (C). Controls without 5-AVD and without cells were included. Figure S4: Effect of heterologous GABA permeases on 5-AVD and l-lysine secretion of *C. glutamicum*. (A) Production characteristics of AVD-2A (*P*_*tuf*_ *davB*), AVD-3 (*P*_*sod*_^*opt2*^ *davB*), and derivatives AVD-7 A/7B carrying *P*_*tuf*_ *gabP* from *P. putida*. (B) Production characteristics of AVD-2A/2B and derivatives AVD-8 A/8B/9A/9B expressing *gabP* variants from *M. smegmatis* or *E. coli*. Cultivations were performed in baffled shake flasks (A) or mini-bioreactors (B) on glucose minimal medium at 30 °C. (C) qRT-PCR verification of heterologous *gabP* expression for one representative from each donor organism. Data represent mean ± SD from three biological replicates. Figure S5: Fed-batch cultivation of strain AVD-2A for 5-AVD production. Fermentation was conducted in sucrose–molasses medium at 30 °C. Substrate is shown as total sugar (sucrose, glucose, fructose). After depletion of initial sugar, concentrated feed pulses were triggered automatically when dissolved oxygen exceeded 40%. Data represent mean ± deviation of two replicates. Figure S6: Verification of the genetic stability of *davB* expression cassettes in 5-AVD producing *C. glutamicum*. PCR products flanking the *davB* integration site were analyzed for AVD-2A and AVD-11. Samples were taken from inoculum (t₀) and final fermentation broth (A, B). Expected amplicon sizes: 3835 bp (AVD-2A) and 3827 bp (AVD-11).

## Data Availability

All data supporting the conclusions of this article are included in this article and its supplementary information.
