# Supplementary material for: Metabolic engineering of Corynebacterium glutamicum for high-yield de novo biosynthesis of 5-aminovaleramide, a promising bio-based monomer
Source: Microb Cell Fact. 2026 Feb 2;25:54. doi: 10.1186/s12934-026-02922-1 (PMC12930585; doi:10.1186/s12934-026-02922-1)
Supplement: Supplementary file 1 — Supplementary Material 1. Table S1: Kinetics and stoichiometry of growth and product formation in C. glutamicum strains producing 5-aminovaleramide (5-AVD) with and without deletion of the l-lysine exporter lyse. Strains were based on the expression of native or codon-optimized davB from P. putida KT2440 under the constitutive promoter Ptuf in chassis strains LYS-1 and LYS-12. Cultivations were performed in mini-bioreactors on glucose minimal medium at 30 °C. Data include specific growth rates (µ) and yields (Y). Data represent mean ± standard error of three biological replicates. Table S2: Intracellular amino acid concentrations in 5-AVD-producing strains AVD-2A and AVD-2B, and their lysE-deficient derivatives AVD-6 A and AVD-6B. Strains were cultivated in baffled shake flasks on glucose minimal medium at 30 °C, and samples were taken during mid-exponential phase. Data represent mean ± standard error of three biological replicates. Table S3. Kinetics and stoichiometry of growth and product formation in C. glutamicum AVD-12, overexpressing lysE under the constitutive sod promoter in strain AVD-3. Cultivation was performed in glucose minimal medium at 30 °C in shake flasks. Data include the specific growth rate (µ), substrate consumption and product formation (q), and yields (Y). Data represent mean ± standard error of three biological replicates. Table S4: Primers used in this study. Table S5: Biochemical reaction network used for C. glutamicum AVD-3 and AVD-11. Table S6: Anabolic drain fluxes used for parameter estimation allowing a 10% deviation in biomass composition [84] and yield (Table 3). All data are given as relative flux (%) related to the specific glucose uptake rate. Table S7: Experimentally determined and simulated relative fractions of mass isotopomers. Figure S1: Isotopic labeling analysis of secreted metabolites in strain LYS-12 Ptuf davBAopt cultivated in glucose minimal medium with fully ¹⁵N-labeled ammonium sulfate. Relative isotope abundances of [file 12934_2026_2922_MOESM1_ESM.pdf]

Additional File 1 to

Metabolic engineering of *Corynebacterium glutamicum* for high-yield de novo biosynthesis of 5-aminovaleramide, a promising bio-based monomer

Annalena Sommer, Sarah Pauli, Michael Kohlstedt, Judith Becker, and Christoph Wittmann*

Institute for Systems Biotechnology, Saarland University, Saarbrücken, Germany

*christoph.wittmann@uni-saarland.de, Phone/Fax: +49-681-302-71970/71972, ORCID 0000-0002-7952-985X

Table S1: Kinetics and stoichiometry of growth and product formation in *C. glutamicum* strains producing 5-aminovaleramide (5-AVD) with and without deletion of the L-lysine exporter *lysE*. Strains were based on the expression of native or codon-optimized *davB* from *P. putida* KT2440 under the constitutive promoter P_{tuf} in chassis strains LYS-1 and LYS-12. Cultivations were performed in mini-bioreactors on glucose minimal medium at 30 °C. Data include specific growth rates (μ) and yields (Y). Data represent mean ± standard error of three biological replicates.

	**μ [h^{-1}]**	**$Y_{5\text{-}AVD/GLC}$ [mmol mol^{-1}]**	**$Y_{LYS/GLC}$ [mmol mol^{-1}]**
LYS-1	0.35 ± 0.01	0.0 ± 0.0	78.1 ± 0.6
LYS-1 *ΔlysE*	0.34 ± 0.01	0.0 ± 0.0	0.1 ± 0.0
AVD-4A	0.27 ± 0.01	17.2 ± 1.4	81.8 ± 1.3
AVD-4B	0.32 ± 0.01	0.0 ± 0.0	0.1 ± 0.1
AVD-5A	0.24 ± 0.01	26.6 ± 1.0	77.4 ± 1.2
AVD-5B	0.23 ± 0.00	0.0 ± 0.0	0.1 ± 0.0
AVD-2A	0.13 ± 0.00	284.9 ± 20.1	0.3 ± 0.1
AVD-6A	0.08 ± 0.01	218.9 ± 1.7	0.1 ± 0.0
AVD-2B	0.12 ± 0.01	214.5 ± 19.7	0.2 ± 0.2
AVD-6B	0.12 ± 0.00	170.4 ± 1.7	0.2 ± 0.1

Table S2: Intracellular amino acid concentrations in 5-AVD-producing strains AVD-2A and AVD-2B, and their *lysE*-deficient derivatives AVD-6A and AVD-6B. Strains were cultivated in baffled shake flasks on glucose minimal medium at 30 °C, and samples were taken during mid-exponential phase. Data represent mean ± standard error of three biological replicates.

Amino acid	AVD-2A [mM]	AVD-6A [mM]	AVD-2B [mM]	AVD-6B [mM]
5-AVD	112.1 ± 5.3	90.7 ± 9.7	83.4 ± 14.4	71.3 ± 2.5
L-Lysine	2.8 ± 1.7	4.6 ± 0.4	8.5± 5.2	5.3 ± 0.4
L-Aspartic acid	4.6 ± 0.2	7.4 ± 1.0	6.2 ± 0.9	5.7 ± 0.7
L-Glutamic acid	134.5 ± 25.0	139.6± 12.6	102.8 ± 15.2	115.5 ± 7.4
L-Asparagine	1.0± 0.2	1.0 ± 0.1	0.6 ± 0.1	0.6 ± 0.1
L-Serine	2.9 ± 1.9	1.7 ± 0.1	2.6 ± 1.7	1.9 ± 0.5
L-Glutamine	11.9 ± 0.7	8.8 ± 0.6	11.6 ± 1.3	8.5 ± 0.7
Glycine	3.1 ± 0.5	2.7 ± 0.2	3.3 ± 0.3	2.8 ± 0.3
L-Threonine	1.4 ± 0.2	1.1 ± 0.1	1.4 ± 0.1	1.2 ± 0.1
L-Arginine	5.2 ± 0.2	5.2± 0.4	4.1 ± 0.3	4.8 ± 0.4
L-Alanine	1.0 ± 0.6	0.8 ± 0.0	0.7 ± 0.4	0.8 ± 0.4
L-Tyrosine	0.5 ± 0.1	0.9 ± 0.1	2.1 ± 0.3	2.3 ± 0.2
L-Valine	2.9 ± 0.2	2.1± 0.6	2.8 ± 1.3	2.0 ± 0.7
L-Methionine	2.6 ± 0.9	1.5 ± 0.9	4.6 ± 3.3	2.1 ± 0.7
L-Leucine	2.2 ± 0.4	1.7 ± 0.2	1.6 ± 0.3	1.2 ± 0.1
L-Tryptophan	0.6 ± 0.1	0.5 ± 0.2	0.8± 0.5	0.8 ± 0.1

Table S3. Kinetics and stoichiometry of growth and product formation in *C. glutamicum* AVD-12, overexpressing *lysE* under the constitutive *sod* promoter in strain AVD-3. Cultivation was performed in glucose minimal medium at 30 °C in shake flasks. Data include the specific growth rate (μ), substrate consumption and product formation (q), and yields (Y). Data represent mean ± standard error of three biological replicates.

	**AVD-12**
**Rates**	
μ [h^{-1}]	0.08 ± 0.02
q_{GLC} [mmol g^{-1} h^{-1}]	2.70 ± 0.50
$q_{5\text{-}AVD}$ [mmol g^{-1} h^{-1}]	0.47 ± 0.09
q_{LYS} [mmol g^{-1} h^{-1}]	0.29 ± 0.06
**Yields**	
$Y_{X/GLC}$ [g mol^{-1}]	28.1 ± 1.8
$Y_{5\text{-}AVD/GLC}$ [mmol mol^{-1}]	174.9 ± 3.7
$Y_{LYS/GLC}$ [mmol mol^{-1}]	107.1 ± 1.6

Table S4: Primers used in this study.

Primer	Sequence	Application
PR_pClik5a_fwd<br>PR_pClik5a_rev	GTGGCCGACAATCAATGAAGCTATG<br>CCGGAGAACCTGCGTGCAATCCAT	Sequencing plasmids based on *pClik5a MCS*
PR_pClikint_fwd<br>PR_pClikint_rev	ATTGTCTGTTGTGCCCAGTCATAG<br>AATAATAGTGAACGGCAGGT	Sequencing plasmids based on *pClik int sacB*
PR I<br>PR II<br>PR III	GACTAGTTCGGACCTAGGGATTGGCCGTTACCCTGCGAATG<br>GAGCATCGATGTCGACGATTTAGTCAGCCAGGGCAATCG<br>GAGCATCGATGTCGACGATTCAATCCGCCAGGGCGATCG	Construction of *pClik5α* P_{tuf} *davB*^{nat} and *pClik5α* P_{tuf} *davB*^{opt}
PR_ups_bioD_fwd<br>PR_davB_opt_rev<br>PR_davB_nat_rev<br>PR_dws_bioD_fwd_(opt)<br>PR_dws_bioD_fwd_(nat)<br>PR_dws_bioD_rev	GACTAGTTCGGACCTAGGGATTAAGCAATGGCCTACAACCA<br>CGAAGGCACGGTGTTCACGATTAGTCAGCCAGGGCAATCG<br>CGAAGGCACGGTGTTCACGATCAATCCGCCAGGGCGATCG<br>CGATTGCCCTGGCTGACTAATCGTGAACACCGTGCCTTCG<br>CGATCGCCCTGGCGGATTGATCGTGAACACCGTGCCTTCG<br>AGAGCATCGATGTCGACGATACGCATGAGTGTGCTTGTGG	Construction of *pClik int sacB* P_{tuf} *davB*$^{Pput\text{-}nat}$ (*bioD*) and *pClik int sacB* P_{tuf} *davB*$^{Pput\text{-}opt}$ (*bioD*)
PR_ups_bioD_fwd<br>PR_ups_bioD_fwd<br>PR_psod_fwd<br>PR_psod_rev<br>PR_davB_fwd<br>PR_davB_rev<br>PR_dws_bioD_fwd_(nat)<br>PR_dws_bioD_rev	GACTAGTTCGGACCTAGGGATTAAGCAATGGCCTACAACCA<br>CCCGGAATAATTGGCAGCTAGGTTTATTTCCCTTTAACTG<br>AGTTAAAGGGAAATAAACCTAGCTGCCAATTATTCCGGG<br>GGCGGTTCTTCTTGTTCATGGGTAAAAAATCCTTTCGTAG<br>ACGAAAGGATTTTTTACCCATGAACAAGAAGAACCGCC<br>CGAAGGCACGGTGTTCACGATCAATCCGCCAGGGCGATCG<br>CGATCGCCCTGGCGGATTGATCGTGAACACCGTGCCTTCG<br>AGAGCATCGATGTCGACGATACGCATGAGTGTGCTTGTGG	Construction of *pClik int sacB* P_{sod} *opt*^{2} *davB*$^{Pput\text{-}nat}$ (*bioD*)
PR_ups_lysE_fwd<br>PR_ups_lysE_rev<br>PR_psod_fwd<br>PR_psod_rev<br>PR_lysE_fwd<br>PR_lysE_rev	ACTAGTTCGGACCTAGGGATGATTAGCTTCACGGGTTACC<br>CCCGGAATAATTGGCAGCTACGTGACCTATGGAAGTACTT<br>AAGTACTTCCATAGGTCACGTAGCTGCCAATTATTCCGGG<br>AAGATTTCCATGATCACCATGGGTAAAAAATCCTTTCGTA<br>TACGAAAGGATTTTTTACCCATGGTGATCATGGAAATCTT<br>AGAGCATCGATGTCGACGATATAAACACAAACGCGTCCAA	Construction of *pClik int* P_{sod} *lysE*
PR_seq_bioD_locus_fwd<br>PR_seq_bioD_locus_rev	AACTCCTCAATCTCACTGG<br>GTGACATTATCACCGTGGTG	Ampflification of *bioD* and flanking areas
qPCR_HK_sigA_fwd<br>qPCR_HK_sigA_rev	GTCTGATTCGTGCCGTAG<br>GGATGGTTCGTGCTTGG	qPCR primer *sigA* [1]
qPCR_gabP_Msm_fwd<br>qPCR_gabP_Msm_rev	TCCTCTTCTACGTCGGGTCG<br>ACGTACGGACTGGACAACAC	qPCR primer *gabP M. smegamatis*
qPCR_gabP_Ecol_fwd<br>qPCR_gabP_Ecol_rev	CCAACTCGGTTATCTGGCGT<br>ACCGAGCGATAAGAACCGAC	qPCR primer *gabP E. coli*
qPCR_gabP_Pput_fwd<br>qPCR_gabP_Pput_rev	GAGGCGGGCAAGCATATTTC<br>AGCACCGTGACATAAGAGCC	qPCR primer *gabP P. putida*
qPCR_davB_fwd<br>qPCR_davB_rev	GACTCGGACTTCCCCAACTC<br>AGATGCCTTGTGGCACTTGT	qPCR primer native *davB*

Table S5: Biochemical reaction network used for *C. glutamicum* AVD-3 and AVD-11.

**Reaction**	**Gene**
GLC_EX + PEP = G6P + PYR	*ptsGHI*
G6P = F6P	*pgi*
G6P = TRE	*treYZ, otsA*
F6P = FBP	*pfk, fbp*
FBP = DHAP + G3P	*fba*
DHAP = G3P	*tpiA*
G6P = Ru5P + CO2	*zwf, pgl, gnd*
Ru5P = X5P	*rpe*
Ru5P = Ri5P	*rpi*
Ri5P + X5P = S7P + G3P	*tkt*
S7P + G3P = E4P + F6P	*tal*
E4P + X5P = F6P + G3P	*tkt*
G3P = 3PG	*gapA, pgk,* ***gapN***
3PG = PEP	*pgm, eno*
PEP = PYR	*pyk*
PYR = ACCOA + CO2	*pdhA, aceEF, lpd*
ACCOA + OAA = CIT	*gltA*
CIT = ICIT	*acn*
ICIT = AKG + CO2	*icd*
AKG = SUCOA + CO2	*odhA, aceEF, lpd*
SUCOA = SUC	*sucCD*
ICIT = SUC + GLYOX	*aceA*
ACCOA + GLYOX = OAA	*glcB*
SUC = FUM	*sdhABCD*
FUM = MAL	*fum*
MAL = OAA	*mdh, mqo*
PYR + CO2 = OAA	*pyc*
MAL = PYR + CO2	*malE*
OAA = PEP + CO2	*pck*
OAA + PYR = LYS + CO2	*aspB, lysC, asd, dapAB, ddh, lysA*

LYS = AVD + CO2	*davB*
LYS = LYS_EX	*lysE*
AVD = AVD_EX	
TRE = TRE_EX	
CO2 = CO2_EX	*CO2 xch*
G6P_B + F6P_B + Ri5P_B + E4P_B + G3P_B + 3PG_B + PYR_B + ACCOA_B + AKG_B + OAA_B = BIOMASS	*BIOMASS*

Table S6: Anabolic drain fluxes used for parameter estimation allowing a 10% deviation in biomass composition [2] and yield (Table 3). All data are given as relative flux (%) related to the specific glucose uptake rate.

	AVD3		AVD11	
	**mean**	**SD**	**mean**	**SD**
G6P_B	0.662	±0.094	0.726	±0.103
F6P_B	0.995	±0.141	1.090	±0.154
Ri5P_B	2.839	±0.402	3.112	±0.440
E4P_B	0.866	±0.122	0.949	±0.134
G3P_B	0.417	±0.059	0.457	±0.065
3PG_B	4.183	±0.592	4.585	±0.648
PEP_B	2.107	±0.298	2.309	±0.327
PYR_B	8.414	±1.190	9.222	±1.304
ACCOA_B	10.263	±1.451	11.248	±1.591
AKG_B	3.954	±0.559	4.333	±0.613
OAA_B	5.427	±0.767	5.948	±0.841

C. Wittmann, A.A. De Graaf „Metabolic flux analysis in *Corynebacterium glutamicum*" L. Eggeling, M. Bott (Eds.), Handbook of *Corynebacterium glutamicum*, CRC Press, Boca Raton (2005), pp. 277-304

Table S7: Experimentally determined and simulated relative fractions of mass isotopomers.

			AVD3		AVD11	
			EXP	SIM	EXP	SIM
99% [1-^{13}C] glucose	Ala 260	m	0.568	0.568	0.529	0.529
		m+1	0.310	0.310	0.340	0.341
		m+2	0.096	0.096	0.102	0.102
		m+3	0.025	0.025	0.029	0.029
	Ala 232	m	0.595	0.599	0.555	0.561
		m+1	0.312	0.309	0.346	0.342
		m+2	0.093	0.092	0.099	0.097
	Gly 246	m	0.748	0.749	0.746	0.745
		m+1	0.179	0.179	0.181	0.182
		m+2	0.073	0.072	0.073	0.073
	Gly 218	m	0.822	0.822	0.821	0.822
		m+1	0.178	0.178	0.179	0.178
	Val 288	m	0.432	0.426	0.371	0.370
		m+1	0.365	0.370	0.390	0.392
		m+2	0.148	0.149	0.173	0.173
		m	0.043	0.044	0.051	0.051
		m+3	0.010	0.010	0.012	0.012
		m+4	0.002	0.002	0.003	0.002
	Val 260	m	0.437	0.440	0.375	0.384
		m+1	0.366	0.368	0.391	0.392
		m+2	0.146	0.143	0.171	0.166
		m+3	0.042	0.040	0.051	0.047
		m+4	0.010	0.009	0.012	0.011
	Leu 274	m	0.351	0.342	0.282	0.278
		m+1	0.378	0.384	0.386	0.390
		m+2	0.190	0.193	0.227	0.228
		m+3	0.062	0.063	0.080	0.080
		m+4	0.015	0.016	0.021	0.021
		m+5	0.003	0.003	0.005	0.004
	Leu 200	m	0.385	0.380	0.308	0.309
		m+1	0.389	0.398	0.402	0.410
		m+2	0.171	0.171	0.215	0.211
		m+3	0.044	0.042	0.061	0.058
		m+4	0.008	0.007	0.012	0.011
		m+5	0.002	0.001	0.003	0.001
	Ile 274	m	0.365	0.365	0.316	0.314
		m+1	0.375	0.379	0.385	0.390
		m+2	0.181	0.181	0.206	0.206
		m+3	0.059	0.058	0.070	0.069
		m+4	0.015	0.014	0.019	0.017
		m+5	0.004	0.003	0.005	0.003
	Ile 200	m	0.407	0.405	0.351	0.349
		m+1	0.386	0.391	0.402	0.407
		m+2	0.158	0.158	0.186	0.187
		m+3	0.039	0.038	0.048	0.048
		m+4	0.008	0.007	0.010	0.009
		m+5	0.003	0.001	0.003	0.001

	Pro 258	m	0.436	0.427	0.375	0.373
		m+1	0.366	0.370	0.391	0.392
		m+2	0.146	0.150	0.172	0.173
		m+3	0.042	0.043	0.051	0.051
		m+4	0.010	0.009	0.012	0.012
	Met 320	m	0.325	0.327	0.277	0.278
		m+1	0.351	0.364	0.355	0.371
		m+2	0.193	0.200	0.213	0.223
		m+3	0.079	0.079	0.092	0.091
		m+4	0.030	0.024	0.036	0.029
		m+5	0.022	0.006	0.028	0.007
	Met 292	m	0.356	0.355	0.309	0.306
		m+1	0.357	0.365	0.368	0.376
		m+2	0.185	0.188	0.206	0.211
		m+3	0.076	0.071	0.086	0.082
		m+4	0.026	0.021	0.030	0.025
	Met 218	m	0.389	0.394	0.342	0.339
		m+1	0.359	0.375	0.376	0.391
		m+2	0.162	0.166	0.185	0.192
		m+3	0.069	0.052	0.074	0.062
		m+4	0.021	0.013	0.022	0.016
	Ser 390	m	0.501	0.500	0.466	0.465
		m+1	0.324	0.325	0.347	0.348
		m+2	0.132	0.132	0.140	0.140
		m+3	0.043	0.043	0.048	0.047
	Ser 362	m	0.533	0.532	0.498	0.499
		m+1	0.334	0.334	0.361	0.360
		m+2	0.133	0.134	0.141	0.141
	Ser 288	m	0.578	0.579	0.540	0.542
		m+1	0.319	0.321	0.350	0.351
		m+2	0.103	0.100	0.110	0.107
	Thr 404	m	0.387	0.387	0.353	0.353
		m+1	0.358	0.358	0.369	0.370
		m+2	0.174	0.174	0.187	0.187
		m+3	0.063	0.063	0.070	0.070
		m+4	0.018	0.018	0.021	0.020
	Thr 376	m	0.418	0.425	0.388	0.392
		m+1	0.358	0.355	0.372	0.370
		m+2	0.165	0.164	0.175	0.175
		m+3	0.058	0.057	0.064	0.062
	Phe 336	m	0.341	0.357	0.288	0.301
		m+1	0.351	0.377	0.361	0.385
		m+2	0.172	0.185	0.198	0.213
		m+3	0.060	0.061	0.073	0.075
		m+4	0.018	0.015	0.022	0.020
		m+5	0.027	0.003	0.027	0.004
		m+6	0.011	0.000	0.011	0.001
		m+7	0.011	0.000	0.011	0.000
		m+8	0.005	0.000	0.005	0.000
		m+9	0.004	0.000	0.004	0.000
	Phe 234	m	0.405	0.406	0.343	0.344
		m+1	0.375	0.390	0.390	0.405
		m+2	0.153	0.158	0.182	0.190

		m+3	0.041	0.039	0.053	0.051
		m+4	0.010	0.007	0.013	0.010
		m+5	0.005	0.001	0.007	0.001
		m+6	0.003	0.000	0.004	0.000
		m+7	0.003	0.000	0.005	0.000
		m+8	0.003	0.000	0.004	0.000
	Phe 302	m	0.727	0.728	0.726	0.726
		m+1	0.196	0.196	0.197	0.198
		m+2	0.077	0.076	0.077	0.076
	Asp 418	m	0.387	0.386	0.355	0.353
		m+1	0.356	0.357	0.368	0.369
		m+2	0.175	0.175	0.187	0.188
		m+3	0.064	0.064	0.070	0.070
		m+4	0.018	0.018	0.020	0.020
	Asp 390	m	0.423	0.424	0.393	0.391
		m+1	0.354	0.354	0.369	0.370
		m+2	0.165	0.164	0.176	0.176
		m+3	0.058	0.057	0.063	0.063
	Asp 316	m	0.461	0.467	0.429	0.431
		m+1	0.354	0.356	0.372	0.375
		m+2	0.139	0.138	0.150	0.150
		m+3	0.046	0.040	0.050	0.044
	Glu 432	m	0.306	0.313	0.263	0.270
		m+1	0.361	0.362	0.364	0.366
		m+2	0.213	0.208	0.233	0.229
		m+3	0.087	0.084	0.099	0.096
		m+4	0.027	0.026	0.032	0.031
		m+5	0.007	0.006	0.008	0.008
	Glu 330	m	0.411	0.408	0.351	0.356
		m+1	0.370	0.372	0.391	0.390
		m+2	0.159	0.160	0.186	0.183
		m+3	0.048	0.049	0.058	0.057
		m+4	0.011	0.011	0.015	0.014
	Lys 431	m	0.306	0.290	0.265	0.247
		m+1	0.353	0.362	0.355	0.362
		m+2	0.214	0.219	0.234	0.241
		m+3	0.088	0.091	0.101	0.105
		m+4	0.029	0.029	0.034	0.035
		m+5	0.008	0.007	0.009	0.009
		m+6	0.003	0.002	0.003	0.002
	Lys 329	m	0.331	0.349	0.295	0.300
		m+1	0.356	0.379	0.379	0.387
		m+2	0.211	0.190	0.219	0.215
		m+3	0.073	0.063	0.079	0.075
		m+4	0.022	0.016	0.022	0.020
		m+5	0.006	0.003	0.005	0.004
	Arg 442	m	0.242	0.258	0.217	0.223
		m+1	0.338	0.354	0.334	0.350
		m+2	0.232	0.235	0.248	0.253
		m+3	0.110	0.105	0.121	0.118
		m+4	0.044	0.036	0.047	0.042
		m+5	0.022	0.010	0.023	0.012
		m+6	0.011	0.002	0.010	0.003

	His 440	m	0.371	0.386	0.324	0.329
		m+1	0.336	0.349	0.346	0.357
		m+2	0.174	0.176	0.194	0.201
		m+3	0.074	0.065	0.085	0.081
		m+4	0.027	0.018	0.032	0.025
		m+5	0.012	0.004	0.013	0.006
		m+6	0.006	0.001	0.006	0.001
	Tyr 466	m	0.305	0.308	0.255	0.260
		m+1	0.346	0.361	0.344	0.362
		m+2	0.200	0.210	0.217	0.233
		m+3	0.083	0.086	0.096	0.101
		m+4	0.033	0.027	0.042	0.033
		m+5	0.012	0.007	0.016	0.009
		m+6	0.007	0.001	0.011	0.002
		m+7	0.005	0.000	0.007	0.000
		m+8	0.004	0.000	0.005	0.000
		m+9	0.005	0.000	0.007	0.000
	Tyr 302	m	0.724	0.728	0.722	0.726
		m+1	0.198	0.196	0.199	0.198
		m+2	0.079	0.076	0.079	0.076
	Tre 361	m	0.109	0.101	0.073	0.070
		m+1	0.583	0.585	0.608	0.604
		m+2	0.198	0.197	0.201	0.204
		m+3	0.089	0.091	0.092	0.095
		m+4	0.017	0.020	0.021	0.021
		m+5	0.003	0.005	0.005	0.005
		m+6	0.000	0.001	0.001	0.001

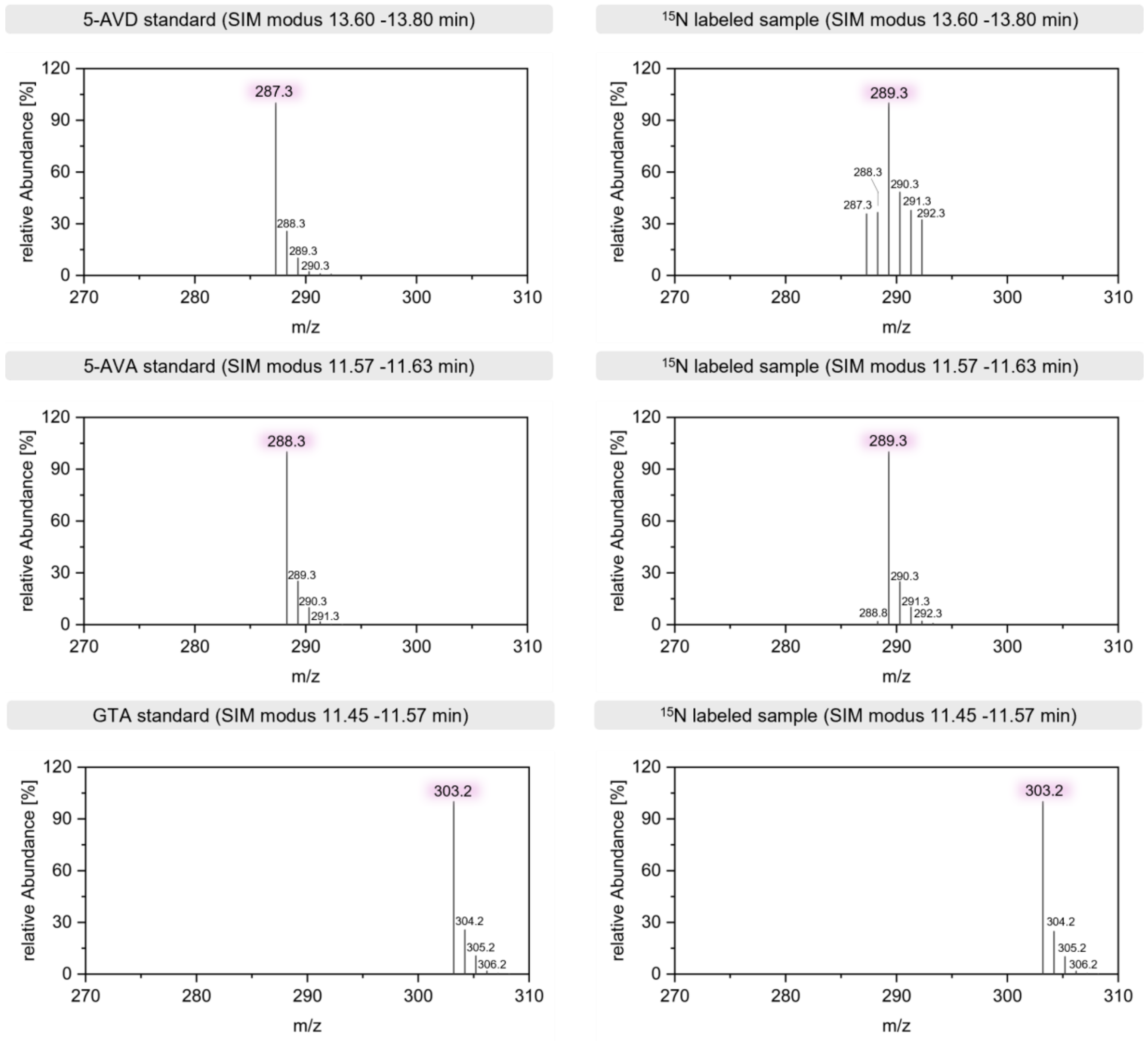

Figure S1: Isotopic labeling analysis of secreted metabolites in strain LYS-12 *P_{tuf}* *davBA^{opt}* cultivated in glucose minimal medium with fully ^{15}N-labeled ammonium sulfate. Relative isotope abundances of major fragment ions ($[M–57]^+$) were compared with commercial standards of 5-AVD, 5-AVA, and GTA. Mass shifts confirmed incorporation of nitrogen atoms (+2 Da for 5-AVD, +1 Da for 5-AVA, +0 Da for GTA).

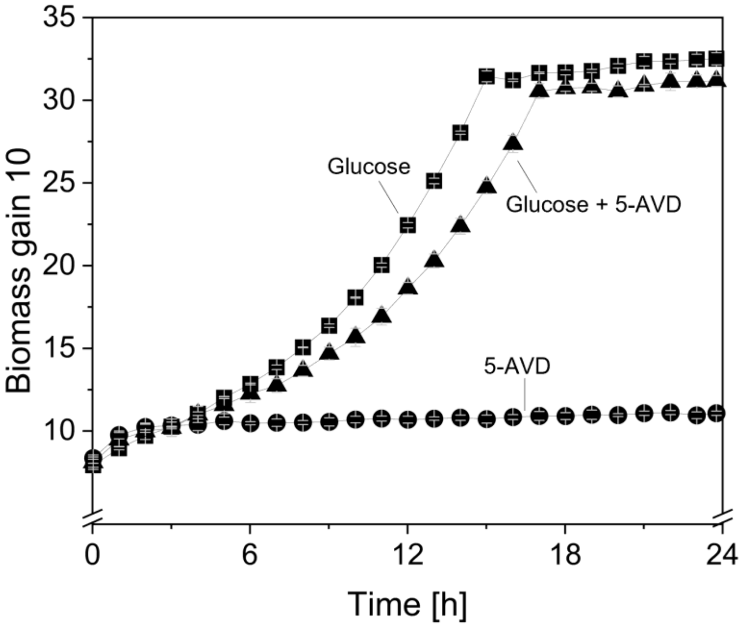

Figure S2: Inability of *C. glutamicum* LYS-12 to grow on 5-AVD as sole carbon source. Growth was monitored in mini-bioreactors with minimal medium containing 10 g L^{-1} glucose, 4.6 g L^{-1} 5-AVD, or both. No biomass formation occurred with 5-AVD alone. Data represent mean ± standard error of three biological replicates.

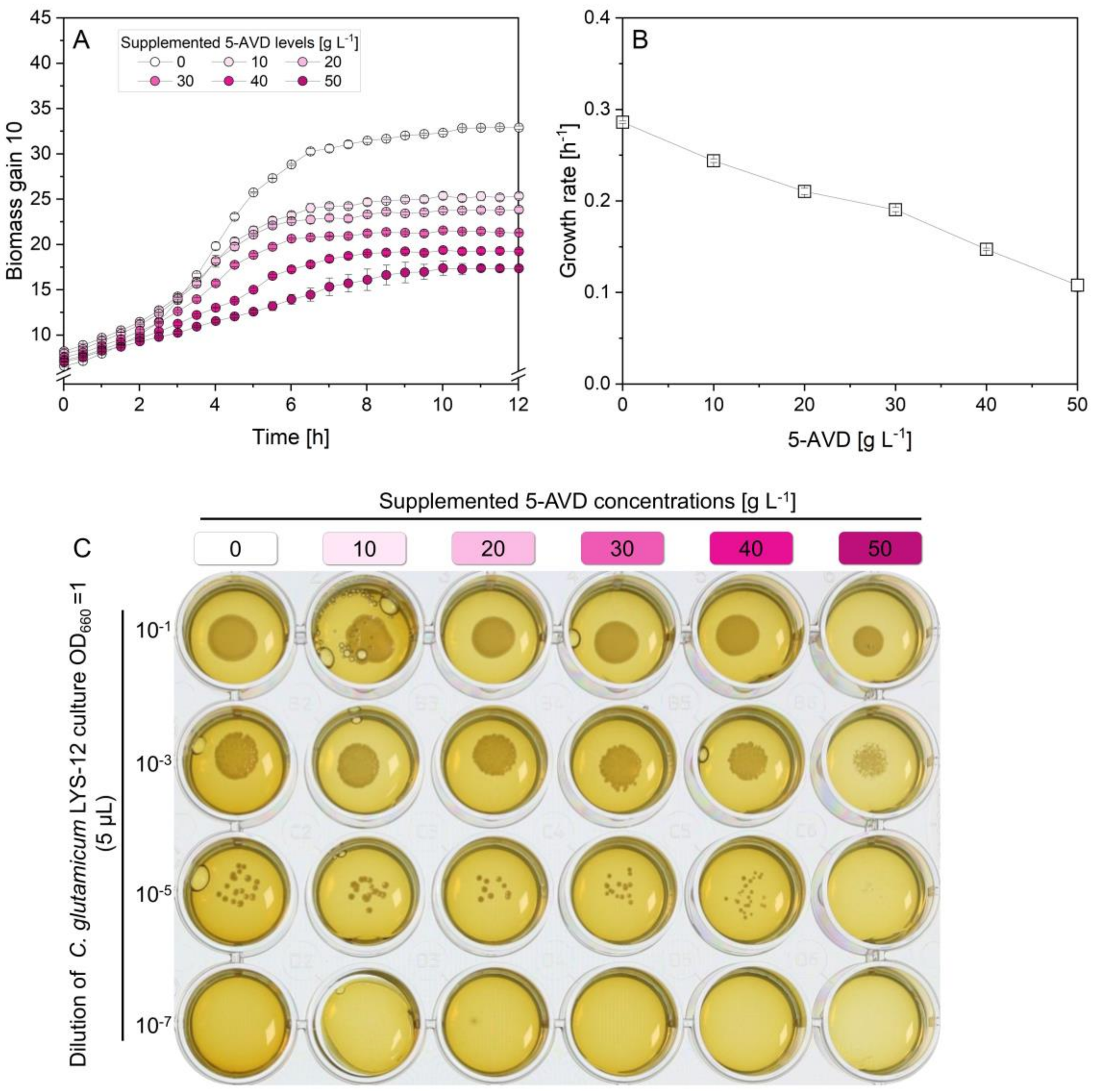

Figure S3: Effect of 5-AVD on the growth of *C. glutamicum* LYS-12. Cells (initial OD_{660} = 1) were cultivated in a miniaturized bioreactor system on BHI medium supplemented with defined 5-AVD concentrations. Data represent mean ± standard error from three biological replicates (A, B). For growth on solid BHI agar, 5 µL of cell dilutions were spotted and incubated at 30 °C for 40 h (C). Controls without 5-AVD and without cells were included.

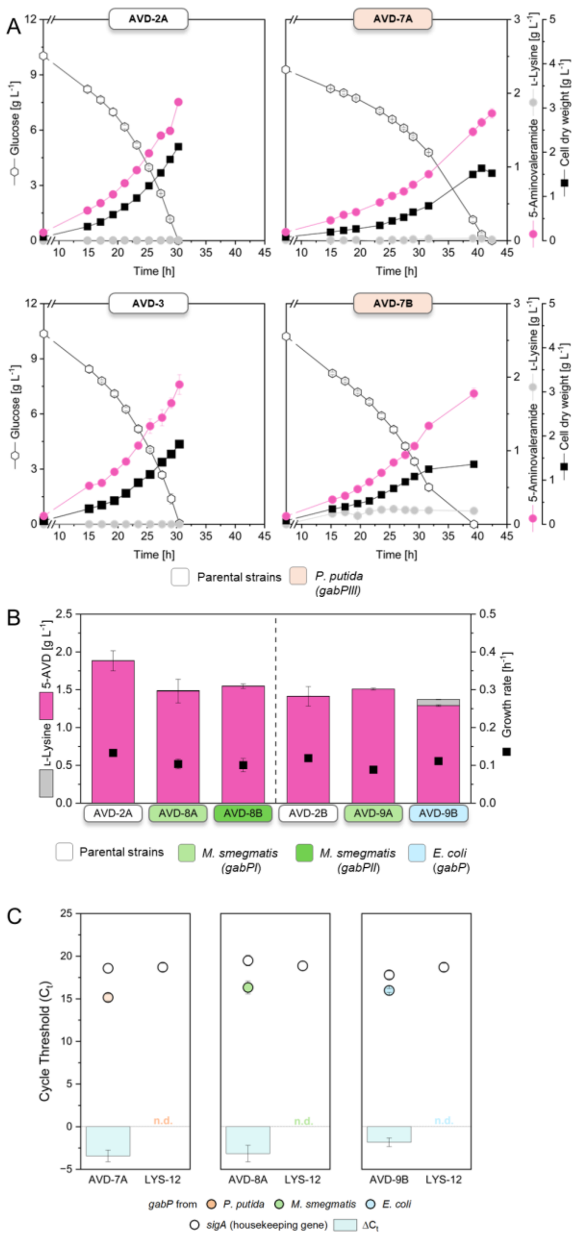

Figure S4: **Effect of heterologous GABA permeases on 5-AVD and L-lysine secretion of *C. glutamicum*.** (A) Production characteristics of AVD-2A (P_{tuf} *davB*), AVD-3 (P_{sod}^{opt2} *davB*), and derivatives AVD-7A/7B carrying P_{tuf} *gabP* from *P. putida*. (B) Production characteristics of AVD-2A/2B and derivatives AVD-8A/8B/9A/9B expressing *gabP* variants from *M. smegmatis* or *E. coli*. Cultivations were performed in baffled shake flasks (A) or mini-bioreactors (B) on glucose minimal medium at 30 °C. (C) qRT-PCR verification of heterologous *gabP* expression for one representative from each donor organism. Data represent mean ± SD from three biological replicates.

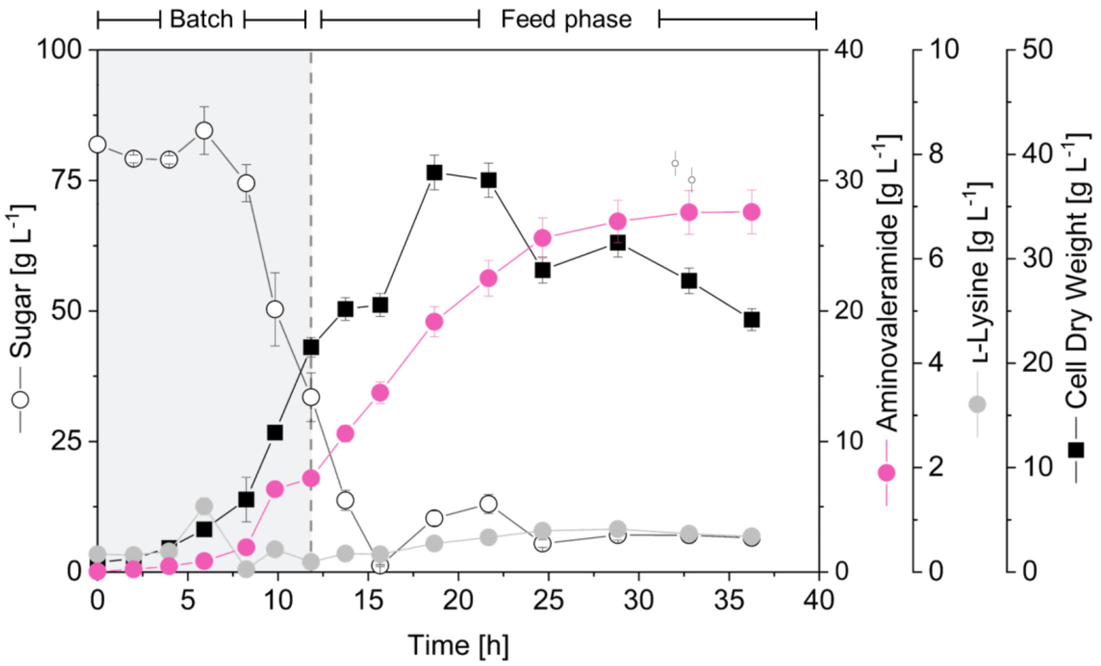

Figure S5: Fed-batch cultivation of strain AVD-2A for 5-AVD production. Fermentation was conducted in sucrose–molasses medium at 30 °C. Substrate is shown as total sugar (sucrose, glucose, fructose). After depletion of initial sugar, concentrated feed pulses were triggered automatically when dissolved oxygen exceeded 40%. Data represent mean ± deviation of two replicates.

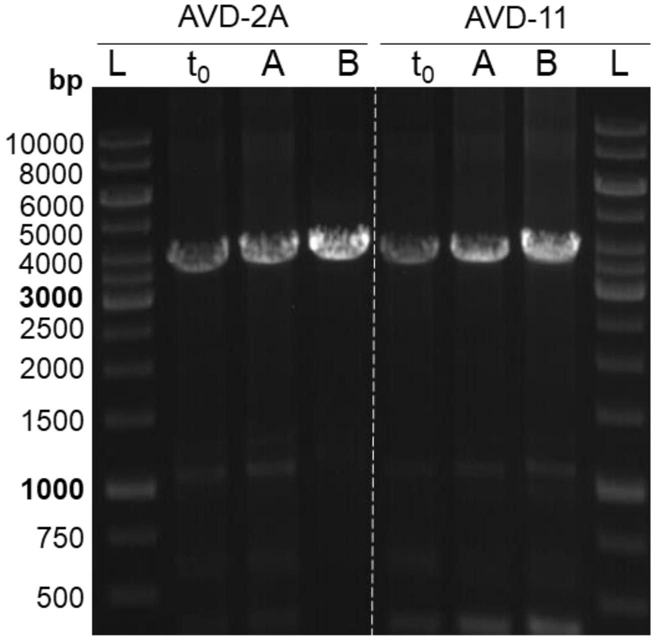

Figure S6: Verification of the genetic stability of *davB* expression cassettes in 5-AVD producing *C. glutamicum*. PCR products flanking the *davB* integration site were analyzed for AVD-2A and AVD-11. Samples were taken from inoculum (t_0) and final fermentation broth (A, B). Expected amplicon sizes: 3835 bp (AVD-2A) and 3827 bp (AVD-11).

References

1. Wang XY, Peng F, Dong GB, Sun Y, Dai XF, Yang YK, Liu XX, Bai ZH: **Identification and validation of appropriate reference genes for qRT-PCR analysis in *Corynebacterium glutamicum*.** *Fems Microbiol Lett* 2018, **365**.
2. Wittmann C, De Graaf AA: **Metabolic flux analysis in *Corynebacterium glutamicum*.** In *Handbook of Corynebacterium glutamicum.* Edited by Eggeling L, Bott M. Boca Raton: CRC Press; 2005: 277-304.
